# Storage Temperature and Moisture Content Differentially Affect Seed Viability, Biochemical Composition, and Oxidative Status in *Lallemantia* Seeds

**DOI:** 10.3390/plants15213435

**Published:** 2026-11-04

**Authors:** Arezoo Paravar, Ramin Piri, Ademola Emmanuel Adetunji, Farzin Abdollahi

**Affiliations:** 1 Horticulture Science Department, Faculty of Agriculture and Natural Resources, University of Hormozgan, Bandar Abbas 79161-93145, Iran; 2 Department of Agronomy and Plant Breeding, Faculty of Agriculture, University of Tehran, Karaj 77871-31587, Iran; raminpiri88@yahoo.com; 3 Plant Science Laboratory, Cranfield University, Cranfield MK43 0AL, UK; adetunjiademola@hotmail.com

**Keywords:** fatty acids, hydrogen peroxide, glucose, tocopherol, total free amino acids

## Abstract

*Lallemantia* species are oilseed crops grown primarily for their seeds; therefore, maintaining the quality of their seeds is crucial for agricultural productivity. However, declining seed quality constitutes a critical challenge to seed sector stakeholders. Prolonged storage duration, elevated storage temperature, and increased seed moisture content are key factors influencing quality loss. We investigated physiological and biochemical processes associated with seed deterioration in *L. iberica* and *L. royleana* seeds stored under different temperatures (15 °C, 35 °C, and 45 °C), moisture contents (5%, 9%, 13%, and 17%), and storage durations (0, 30, 60, and 90 days). Seed viability progressively declined with increasing storage temperature, seed moisture content, and storage duration, reaching 2% in *L. iberica* and 14% in *L. royleana* under the most severe storage conditions (45 °C, 17% seed moisture content, and 90 days of storage). Gradual compromise of membrane integrity was associated with increased electrical conductivity (EC), ROS (H_2_O_2_ and O_2_•^−^), and malondialdehyde (MDA) content. Seed deterioration was also characterized by a compromised antioxidant defense system and degradation of seed storage compounds. We observed negative correlations between EC, pH, H_2_O_2_, O_2_•^−^, MDA, glucose, and fructose and germination rate, viability, antioxidant system, and seed storage compounds. *Lallemantia iberica* seeds exhibited higher levels of glucose, fructose, EC, H_2_O_2_, O_2_•^−^, and MDA, as well as greater susceptibility to adverse storage conditions than those of *L. royleana*, as evidenced by greater reductions in viability, germination rate, pH, antioxidant system, and seed storage compounds. Among the storage conditions evaluated, seeds stored at 5% seed moisture content and 15 °C for up to 30 days generally maintained higher viability, germination rate, vigor, antioxidant activity, and seed storage compounds, along with lower levels of membrane damage. However, the suitability of storage conditions was species- and duration-specific, as *L. royleana* maintained 83% viability at 5% moisture content and 35 °C after 90 days. The findings indicate that unfavorable storage conditions induce oxidative stress, membrane damage, weakened antioxidant defenses, and degradation of storage reserves, ultimately reducing seed viability, germination, and vigor.

## 1. Introduction

Seeds, as crucial units of propagation, play a vital role in the evolution and survival of higher plants [1]. Seedling establishment represents a critical and sensitive stage in crop production. Seedlings with higher vigor exhibit enhanced tolerance to both biotic and abiotic stresses, making seed vigor an essential factor for achieving optimal crop yields [2]. In agriculture, high-quality seeds are a fundamental prerequisite for uniform germination and seedling growth, and ultimately improved economic returns [3]. In recent years, noticeable changes in climatic conditions have exacerbated challenges with seedling establishment, underscoring the critical importance and urgency of using high-quality seeds [4,5]. Consequently, successful agricultural production depends strongly on the availability of high-quality seed [6]. Seed quality and vigor are not only for their effective use in food consumption, but also play a fundamental role in long-term storage [1]. Like other plant organs, seeds age naturally over time, even under optimal storage conditions. This aging process gradually reduces seed vigor and viability until eventual death [7].

Seed aging represents an inevitable and irreversible deterioration process, driven by both intrinsic and extrinsic factors [8]. It is manifested through a series of physical, physiological, and biochemical changes that progressively reduce germination capacity and seedling establishment, making it a significant challenge for both domestic and international seed trade [9]. Seed deterioration is driven by various factors, including genetic characteristics, mechanical damage, moisture and oil contents, temperature fluctuations, relative humidity, pest infestation, and oxygen availability. However, variables such as temperature, relative humidity, seed moisture content, and storage duration are particularly influential in determining the rate of seed aging in storage [10]. While ambient storage environments, usually around 25 °C, may allow for relatively gradual deterioration, assessing seed responses across distinct temperature levels can provide greater insight into seed structural stability as well as physiological and biochemical resilience. For instance, elevated temperatures can substantially accelerate deterioration and are therefore often used in artificial aging investigations to create storage stress and demonstrate differences in seed longevity [11,12]. Conversely, low temperatures slow down metabolic deterioration and can substantially increase longevity [13]. Therefore, to reduce storage-related losses, it is crucial to understand how seeds respond to distinct environmental conditions and storage duration [14].

A key process underlying seed aging is oxidative stress, in which the production and accumulation of reactive oxygen species (ROS), such as hydrogen peroxide (H_2_O_2_), superoxide anion (O_2_^•–^), and hydroxyl radicals (^•^OH), exceed the capacity of the antioxidant defense system [6]. The accumulation of ROS causes cellular deterioration by compromising membrane integrity, denaturing proteins, degrading nucleic acids, and destroying cytoplasmic organelles [15]. Seed aging is frequently initiated by membrane damage, as loss of membrane integrity causes the leakage of cytoplasmic contents into intercellular spaces [16]. This membrane degradation is mainly induced by lipid peroxidation and auto-oxidation, leading to key alterations, such as enhanced solute leakage, elevated free fatty acid levels, loss of membrane phospholipids, decreased tocopherol content, and impaired adenosine triphosphate (ATP) synthesis, all of which progressively diminish seed vigor and viability [7,17]. It has been demonstrated that in species such as *Avena sativa* L. [18], *Glycine max* L. [19], and *L. iberica* [20], seed aging increases membrane permeability, which in turn increases electrical conductivity. In addition, it has been reported that loss of seed viability can be due to the accumulation of lipid peroxidation products, such as MDA [1,21].

Several studies have reported that oxidative stress arises when ROS production in cells, tissues, or organs exceeds the neutralizing capacity of the antioxidant defense system. This imbalance leads to cellular damage and disruption of physiological functions [15,16]. Enzymatic and non-enzymatic antioxidants protect against ROS. They include free radical- and peroxide-scavenging enzymes such as superoxide dismutase (SOD), catalase (CAT), ascorbate peroxidase (APX), dehydroascorbate (DHA), and glutathione reductase (GR), as well as non-enzymatic antioxidants such as ascorbate (vitamin C), tocopherol (vitamin E), thiols, phenolic compounds, and carotenoids [22]. Previous studies have indicated a strong association between antioxidant enzymes and the maintenance of seed vigor and viability [1,17]. However, some reports have shown that seeds stored at high moisture content and temperature conditions exhibit reduced antioxidant enzyme activity [23].

Declines in seed vigor and viability during prolonged storage have been associated with physicochemical deterioration of carbohydrate reserves and changes in starch and soluble sugar contents [1]. Moreover, certain soluble sugars, such as sucrose, raffinose, and trehalose, are considered protective solutes that help preserve membrane and protein stability during storage [24]. Previous studies on *Carthamus tinctorius* L. [25] and *Zea mays* L. [26] have shown that reduced sucrose and raffinose levels during long-term storage may reduce their protective capacity against oxidative and membrane damage. Additionally, the accumulation of reducing sugars can initiate the Maillard reaction, resulting in the production of harmful compounds that disrupt cellular metabolic processes [27].

The rate of seed deterioration is affected not only by the storage conditions but also by the seed’s fundamental chemical composition [1]. Oilseeds, owing to their high fatty acid content and fragile seed coats, are highly sensitive to storage environments [28]. The composition of fatty acids is one of the major factors determining the susceptibility of oils to oxidative degradation [29]. During storage, alterations in fatty acid composition and lipid auto-oxidation lead to a reduction in oil content and germination capacity, thereby diminishing seed longevity [8,30]. These processes are widely recognized as key contributors to accelerated deterioration of oilseed crops during storage [31].

Oilseed crops like dragonhead (*Lallemantia iberica*) and Lady’s mantle *(Lallemantia royleana*) are annual herbs belonging to the *Lamiaceae* family and are cultivated in Southwestern Asia and Europe [16]. *Lallemantia* species are oilseed crops grown mainly for their seeds, which contain approximately 50 to 60% oil. *Lallemantia* seed is an excellent source of quality oil, which is higher in quality than olive oil [32,33]. The commercial value of *Lallemantia* oil relies entirely on seed quality, yet preserving this quality is highly challenging for both domestic and global trade [34]. Aging-induced seed deterioration is a crucial concern for these oilseeds, especially during storage before marketing [35]. Seeds with high oil content, such as *Lallemantia*, are vulnerable to deterioration because of lipid instability [36,37].

Previous studies have investigated different aspects of seed deterioration in *Lallemantia* species, but they differed in the storage factors, experimental contexts, and range of traits examined. Paravar et al. [20] investigated the effects of seed moisture content, storage temperature, and storage duration on physiological and biochemical responses in *L. iberica*, focusing on a single species and a more limited set of traits. Paravar et al. [16] examined the storage behavior of *L. iberica* and *L. royleana* seeds produced under maternal drought stress, comparing different storage and aging environments, including controlled aging, elevated oxygen and nitrogen atmospheres, and natural storage. More recently, Maleki Farahani et al. [37] evaluated the effects of seed moisture content and storage duration on germination and selected biochemical indices in both species.

Although these studies have established that storage conditions influence seed deterioration in *Lallemantia*, an important knowledge gap remains regarding the comparative responses of the two species when seed viability and a broader range of physiological and biochemical traits are evaluated together under systematically varied storage conditions. In particular, it remains unclear how changes in fatty-acid composition, storage carbohydrates, antioxidant defenses, oxidative stress indicators, respiration and oxygen status, acidity, and membrane integrity relate to the maintenance or loss of seed viability across storage temperatures, seed moisture contents, and storage durations. It is also unclear whether the two species exhibit distinct responses under comparable storage conditions. These questions are relevant because *Lallemantia* seeds are rich in unsaturated fatty acids [32], which may be susceptible to oxidative damage during storage.

Therefore, the present study aimed to determine how storage temperature, seed moisture content, and storage duration affect seed viability and quality in *L. iberica* and *L. royleana*. By integrating germination measurements with these physiological and biochemical traits, we sought to characterize the changes associated with seed deterioration, identify storage conditions that better preserve seed viability and quality over time, and compare the responses of the two species. We hypothesized that the maintenance of seed viability would be associated with lower oxidative damage, greater antioxidant capacity, and greater membrane integrity; higher storage temperature, higher seed moisture content, and longer storage duration would generally accelerate deterioration; and that the two species would differ in their responses to these conditions.

## 2. Results

The ANOVA results showed that storage duration, seed moisture content, storage temperature, plant species, and their interactions varied across the measured seed quality, physiological, and biochemical traits. Detailed statistical results for germination indices and seedling growth, oxidative and antioxidant-related traits, and seed storage compounds are presented in Supplementary Tables S1–S3, respectively.

The statistical analysis revealed significant effects of storage moisture content, storage duration, storage temperature, and plant species, as well as several interactions among these factors, on seed germination and other measured seed quality, physiological, and biochemical traits. The LogWorth values for germination are summarized in Table 1, whereas the corresponding statistical results for the other measured traits are provided in Supplementary Tables S4–S8. For germination, storage duration showed the strongest statistical evidence against the null hypothesis (LogWorth = 102.73), followed by storage temperature (69.57), moisture content (63.86), and plant species (29.05). LogWorth values reflect the statistical strength of evidence against the null hypothesis.

### 2.1. Seed Viability

The seed viability model describes and predicts changes in seed viability during storage as a function of seed moisture content, storage temperature, and storage duration. The fitted seed viability equations provided species-specific estimates of inherent seed longevity and responses to storage conditions in *L. iberica* and *L. royleana*. *L. iberica* exhibited a lower species-specific constant for inherent seed longevity (K_E_ = 2.61 ± 0.067) than *L. royleana* (K_E_ = 3.91 ± 0.290), with a statistically significant difference between the estimates (*p* < 0.001). Similarly, C_W_ was significantly lower in *L. iberica* (0.0834 ± 0.035) than in *L. royleana* (1.40 ± 0.154; *p* < 0.001). In contrast, although C_H_ was numerically higher in *L. iberica* (0.032 ± 0.004) than in *L. royleana* (0.0022 ± 0.019), the difference was not statistically significant (*p* = 0.12). Likewise, the difference in C_Q_ between *L. iberica* (0.0008 ± 0.0007) and *L. royleana* (0.0004 ± 0.0003) was not statistically significant (*p* = 0.60) (Table 2). Therefore, the observed differences in KE and CW were statistically supported, whereas the numerical differences in CH and CQ may not be taken as evidence of species-specific differences in temperature sensitivity.

**Figure 1:**
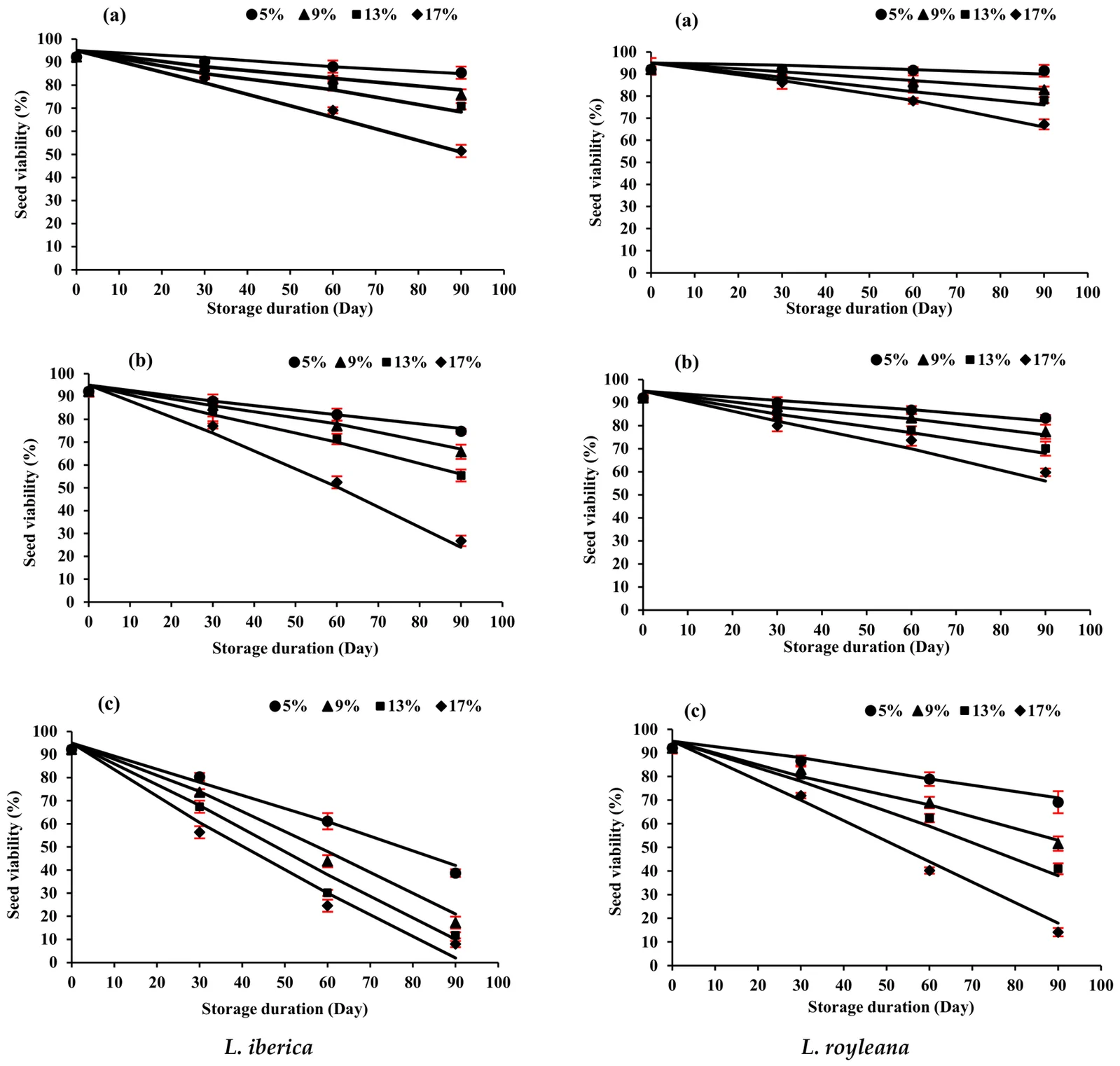
Loss of viability of stored seeds of *L. iberica* (**Left**) and *L. royleana* (**Right**) at 15 °C (**a**), 35 °C (**b**), and 45 °C (**c**) with four moisture content levels for different storage durations. Values are presented as means ± SE (*n* = 3). Error bars represent the standard error of the means.

As storage temperature and moisture content increased over the 0–90-day storage period, viability declined in both *Lallemantia* species. At 15 °C (cool temperature), *L. iberica* seeds stored at 5% moisture content retained 89% viability. At higher moisture levels of 13% and 17%, germination rates declined to 70% and 51%, respectively (Figure 1a). Additionally, *L. royleana* seeds exhibited a comparable response to low-temperature storage, maintaining high viability (85%) at 5% moisture content. Higher moisture content (17% MC) resulted in greater loss of viability (63%) (Figure 1a).

Adjusting the storage temperature to 35 °C (warm temperature) markedly reduced seed viability across all moisture levels, although the extent of viability loss varied substantially with seed moisture content. For *L. iberica* seeds maintained at 5% moisture content, viability remained relatively high at 74% following the 90-day storage period. Further MC elevation (17%) resulted in a more pronounced viability loss, with germination dropping to 26% (Figure 1b). In contrast, *L. royleana* seeds demonstrated a parallel but more pronounced response pattern at 35 °C (warm temperature). At 5% moisture content, this species maintained 81% viability, indicating reasonably good storage stability under these conditions (Figure 1b).

For *L. iberica* seeds at 5% MC, viability declined from 92% to 38% over 90 days at 45 °C (Figure 1c), while viability of *L. royleana* seeds decreased to 69%. The most severe deterioration occurred at 17% MC and 45 °C, where viability plummeted to 2% and 14% in *L. iberica* and *L. royleana*, respectively (Figure 1c).

### 2.2. Prediction of Seed Longevity

**Figure 2:**
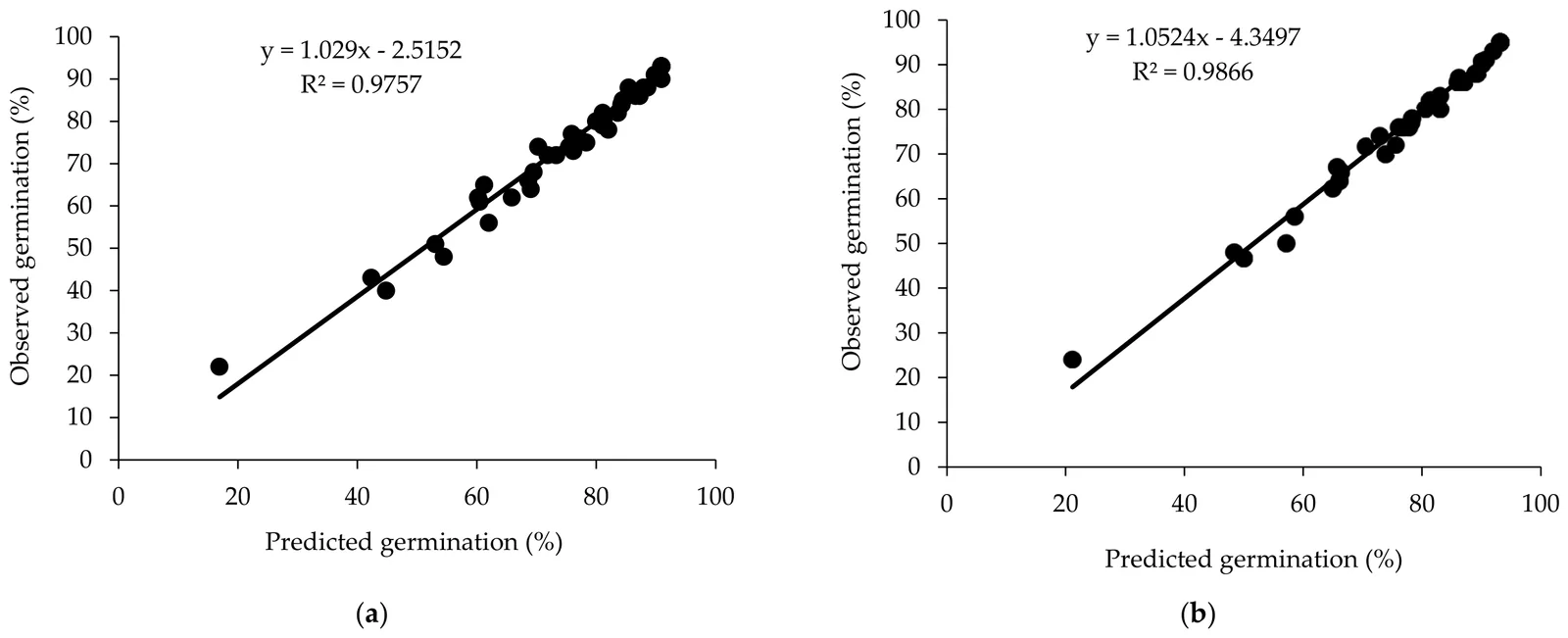
Regression relationship between predicted and observed seed viability values of *L. iberica* (**a**) and *L. royleana* (**b**) under storage conditions.

The seed viability model was used to estimate seed viability under different storage conditions, including storage temperatures and seed moisture contents. The predicted seed viability values from the fitted seed viability model represent the viability estimated for the corresponding combinations of storage temperature, moisture content, and storage duration. The regression analyses showed a strong concordance between the predicted and experimentally observed seed viability for *L. iberica* (Figure 2a) and *L. royleana* (Figure 2b) across a range of storage temperatures and moisture contents. The close clustering of data points along the one-to-one (1:1) line of perfect agreement indicates high accuracy in the model’s predictions. The model’s statistical validity was further supported by the regression parameters. The intercept not significantly differing from zero and the slope not significantly deviating from one confirm that the model’s coefficients were unbiased. The random dispersion of residuals around the regression line, with no systematic pattern, further supports the robustness of the model. This pattern aligns with established principles of seed aging, in which physiological degradation follows predictable kinetics governed by temperature and moisture. Consequently, the model provides a reliable tool for estimating seed longevity in both *Lallemantia* species and may support seed storage and germplasm conservation strategies.

### 2.3. Germination Parameters, Vigor, and Seedling Growth

**Figure 3:**
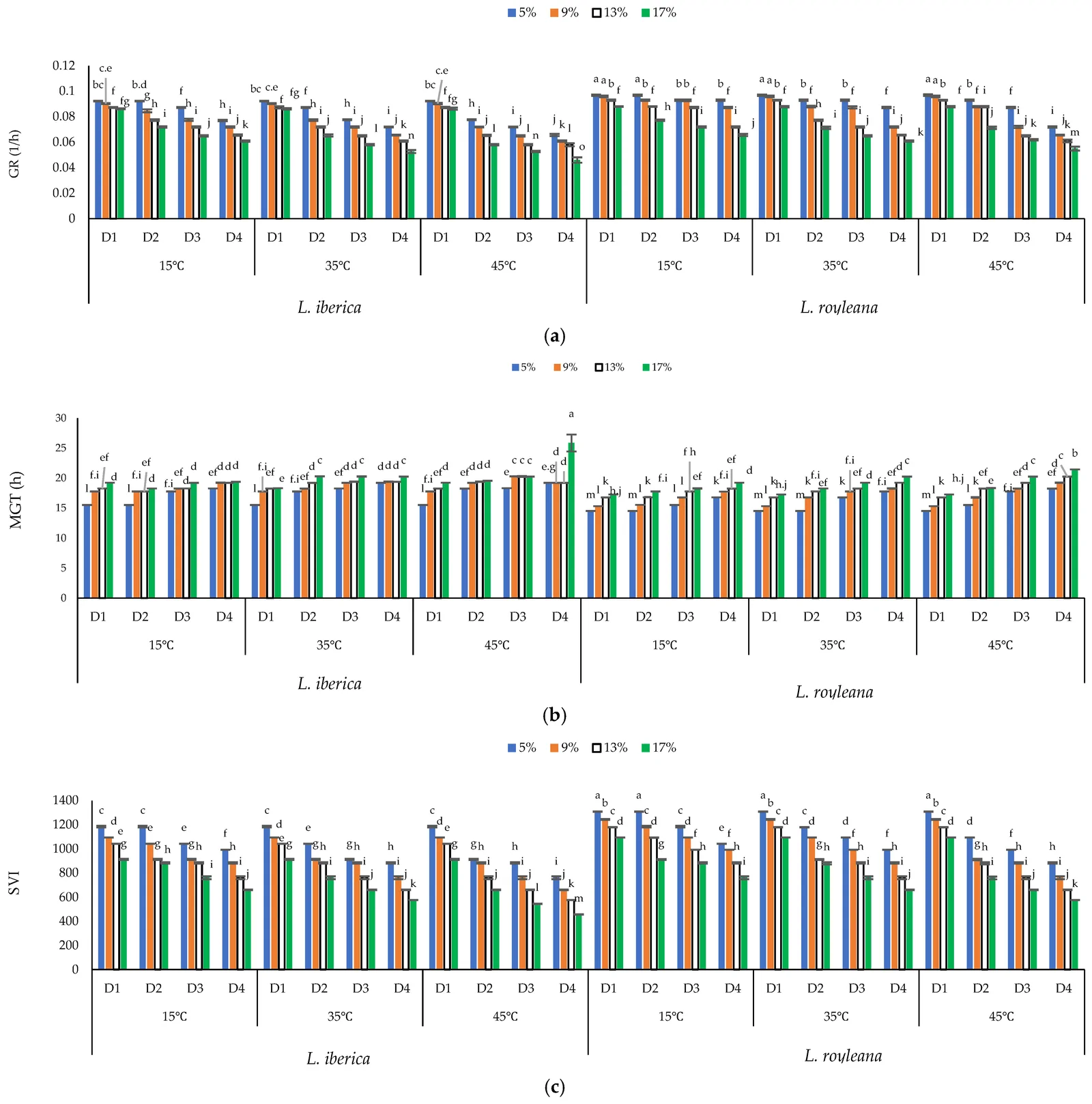
The GR (**a**), MGT (**b**), and SVI (**c**) of *L. iberica* and *L. royleana* under different storage treatments. The bars indicate means ± SEM of three replicates. Different letters indicate significant differences (*p* < 0.05) by Tukey’s multiple range test. For clarity, statistical grouping letters with omitted intermediate letters are presented with a dot (e.g., “a.d” represents “abcd”); the dot has no statistical meaning. GR and MGT were calculated from daily germination counts during the 14-day germination test at 10 °C. Seedling vigor index was determined at the end of the 14-day germination test at 10 °C. Storage duration (D1: 0, D2: 30 days, D3: 60 days, and D4: 90 days after treatment application). GR: germination rate; MGT: mean germination time; SVI: seedling vigor index.

The results showed a significant reduction in germination rate (52%) in seeds stored at 17% MC and 45 °C for over 60 days compared with seeds stored at 5% MC and 15 °C for 0 and 30 days (Figure 3a). The highest and lowest vigor index values (Figure 3b) were observed in seeds stored at 5% and 17% MC, respectively. Seeds stored at 15 °C (cool temperature) showed the highest germination rate. The most pronounced reduction in germination rate and vigor index occurred between 60 and 90 days of aging. Compared with aged *L. iberica* seeds, *L. royleana* seeds showed the highest germination rate (0.097 1/h) and vigor index (1309.9).

The MGT in both *Lallemantia* species improved with higher seed moisture content. The lowest and highest MGT were recorded at 15 °C (cool temperature) and 45 °C (high temperature), respectively. Prolonged storage from 60 to 90 days remarkably increased MGT. The decline in MGT was more pronounced in *L. royleana* (21.43 h) seeds than in *L. iberica* (25.83 h) (Figure 3c).

Seedling length and weight (Table 3) declined with increasing seed moisture content, with seeds stored at 5% MC showing the highest values. Elevated storage temperature further reduced seedling growth. Seedlings from seeds stored for 30 days indicated the maximum length and weight, while prolonged storage led to reductions. Compared to *L. iberica*, the highest seedling growth was observed in aged *L. royleana* seeds.

**Figure 4:**
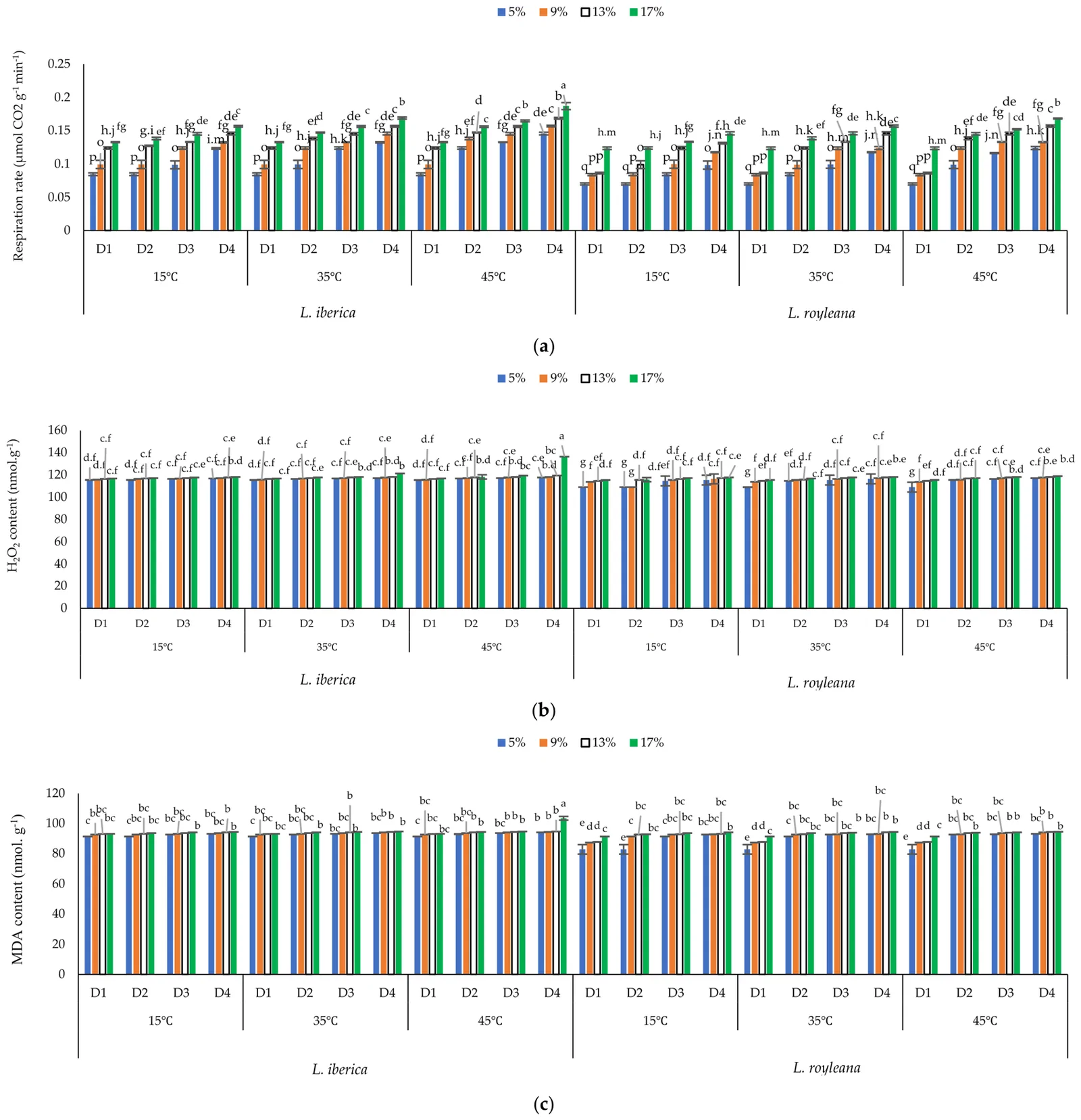
Respiration rate (**a**), H_2_O_2_ level (**b**), and MDA level (**c**) in *L. iberica* and *L. royleana* under different storage treatments. The bars indicate means ± SEM of three replicates. Different letters indicate significant differences (*p* < 0.05) by Tukey’s multiple range test. For clarity, statistical grouping letters with omitted intermediate letters are presented with a dot (e.g., “a.d” represents “abcd”); the dot has no statistical meaning. Respiration rate, H_2_O_2_, and MDA were determined in seeds immediately after the respective storage treatments. Storage duration (D1: 0, D2: 30 days, D3: 60 days, and D4: 90 days after treatment application). H_2_O_2_: hydrogen peroxide; MDA: malondialdehyde.

The lowest respiration rate (Figure 4a) was detected in aged seeds at 5% MC, while seeds with greater moisture content (9% to 17% MC) showed a rising trend in respiration rate. Seeds stored at higher storage temperatures, especially 45 °C, exhibited a greater increase in respiration rate, whereas seeds stored at 15 °C (cool temperature) sustained the lowest rates. Prolonged storage from 0 to 90 days gradually enhanced the respiration rate. *Lallemantia iberica* seeds exhibited a greater respiration rate than *L. royleana*.

### 2.4. Oxidative Products and Antioxidant and Non-Antioxidant Enzymes

As seed moisture content rose from 5% to 17% MC, EC increased and leachate pH declined (Table 4). Increasing storage temperature resulted in higher EC and lower pH, with the maximum EC and minimum pH shown in seeds stored at 45 °C (high temperature). Similar trends were observed over storage durations from 30 to 90 days. Aged *L. iberaca* seeds showed greater EC and lower leachate pH in comparison to *L. royleana*.

Compared to seeds stored at 5% MC, the highest levels of H_2_O_2_ (Figure 4b), O_2_•^−^ (Table 4), and MDA (Figure 4c) were recorded in seeds stored under high moisture content. Elevated storage temperature, especially 45 °C (high temperature), caused greater oxidative stress. Prolonged storage up to 90 days resulted in the greatest H_2_O_2_, O_2_•^−^, and MDA contents compared to shorter storage durations. Aged *L. iberica* seeds indicated higher levels of H_2_O_2_ (Figure 4b), O_2_•^−^ (Table 4), and MDA (Figure 4c) than *L. royleana*.

**Figure 5:**
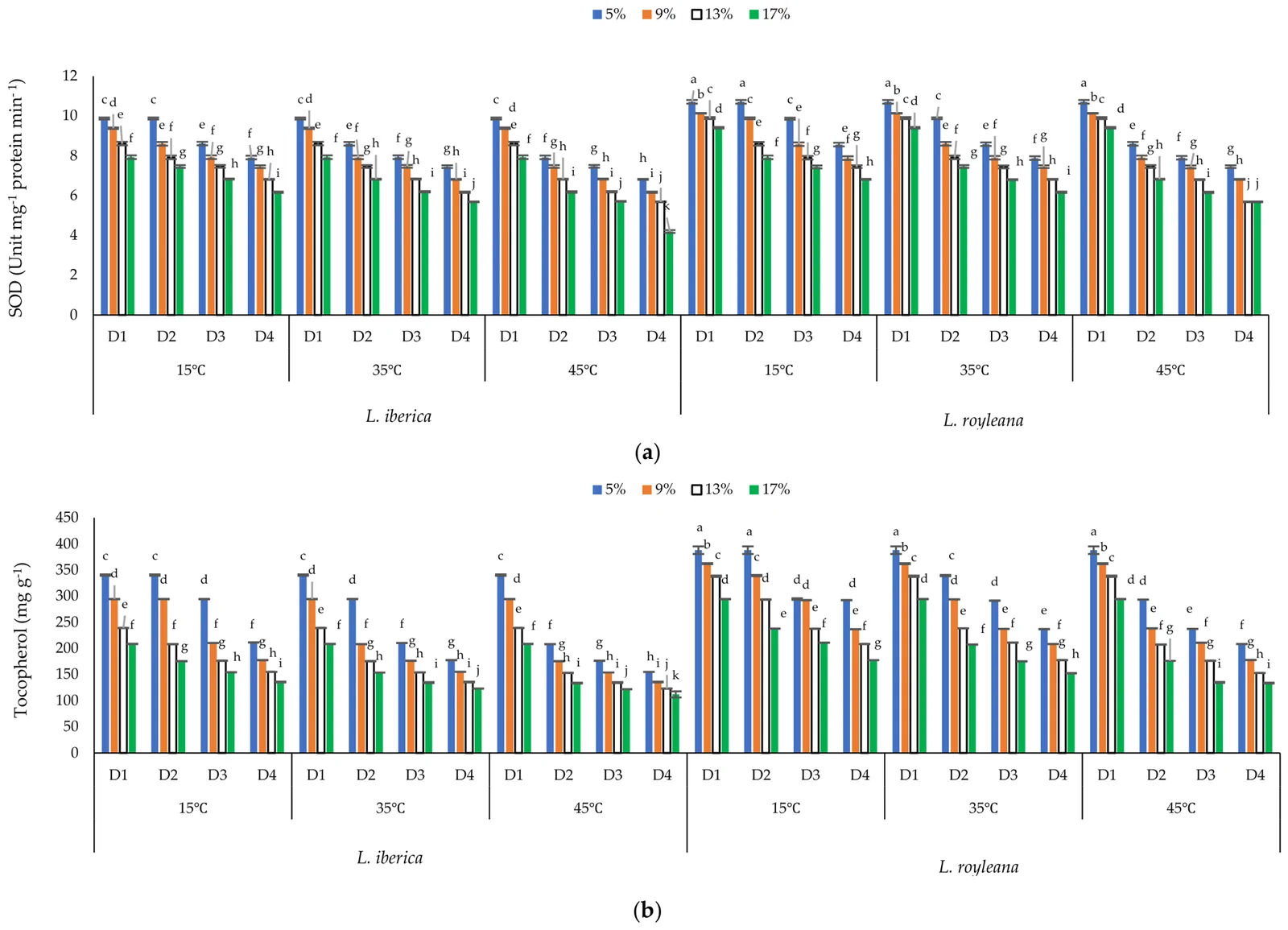
Superoxide dismutase (**a**) and tocopherol (**b**) levels in *Lallemantia* species under different storage treatments. The bars indicate means ± SEM of three replicates. Different letters indicate significant differences (*p* < 0.05) by Tukey’s multiple range test. SOD and tocopherol were determined in seeds immediately after the respective storage treatments. Storage duration (D1: 0, D2: 30 days, D3: 60 days, and D4: 90 days after treatment application). SOD: superoxide dismutase.

The highest antioxidant enzyme activities, such as SOD (Figure 5a), CAT, and APX (Table 5), were exhibited in seeds stored at low moisture content (5% MC), but the activities declined as moisture content increased. Elevated storage temperature reduced the activities of these enzymes, with the maximum and minimum activities obtained at 15 °C and 45 °C. Compared with shorter storage durations, SOD, CAT, and APX activities declined in seeds stored for 60 to 90 days. Aged *L. royleana* seeds exhibited higher antioxidant enzyme activities than *L. iberica*.

A reducing trend in tocopherol content was observed with the rise in seed moisture content. The highest and lowest tocopherol content were obtained in seeds aged at 5% MC and 17% MC, respectively. Compared to storage at cool temperature (15 °C), the lowest tocopherol content occurred in seeds stored at 35 °C and 45 °C. A progressive reduction in tocopherol was recorded over the storage period from 60 to 90 days. Overall, *L. royleana* maintained higher tocopherol content compared to *L. iberica* (Figure 5b).

### 2.5. Seed Storage Compounds

**Figure 6:**
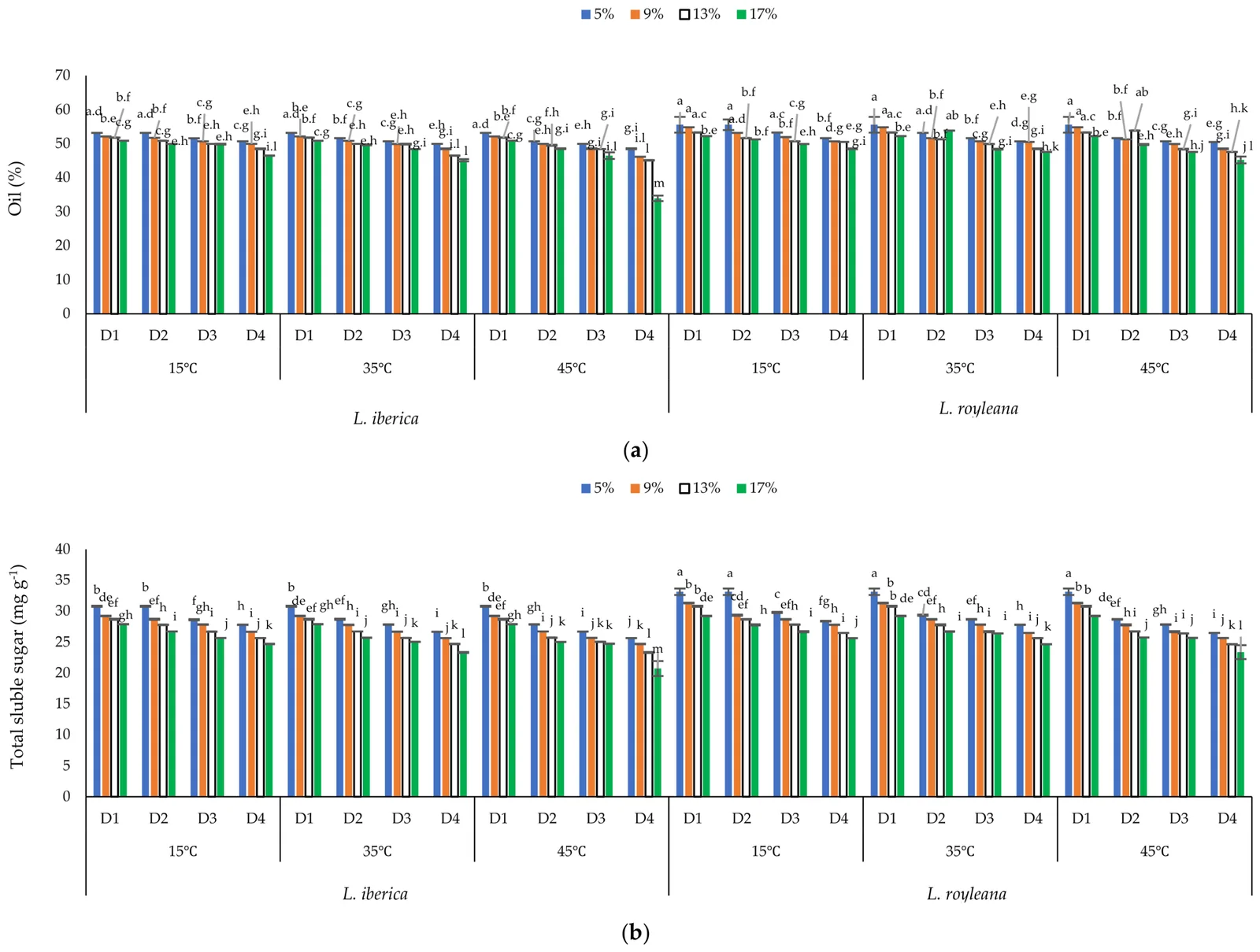
Oil (**a**) and total soluble sugar (**b**) levels in *L. iberica* and *L. royleana* seeds under different storage treatments. The bars indicate means ± SEM of three replicates. Different letters indicate significant differences (*p* < 0.05) by Tukey’s multiple range test. For clarity, statistical grouping letters with omitted intermediate letters are presented with a dot (e.g., “a.d” represents “abcd”); the dot has no statistical meaning. Oil and total soluble sugar were determined in seeds immediately after the respective storage treatments. Storage duration (D1: 0, D2: 30 days, D3: 60 days, and D4: 90 days after treatment application).

According to the results, increasing seed moisture contents led to a remarkable decline in oil content (Figure 6a) and unsaturated fatty acids such as linoleic acid (LNA), linolenic acid (LA), and oleic acid (OA) (Table 6), while palmitic acid (PA) and stearic acid (SA) were enhanced (Table 6). A higher storage temperature caused a remarkable reduction in oil and unsaturated fatty acids, and an increase in saturated fatty acids. Oil and unsaturated fatty acid contents were highest after 0 and 30 days of storage, but reduced markedly after 60 to 90 days. *L. royleana* seeds exhibited higher levels of oil and unsaturated fatty acids, while *L. iberica* seeds had greater saturated fatty acid contents.

**Figure 7:**
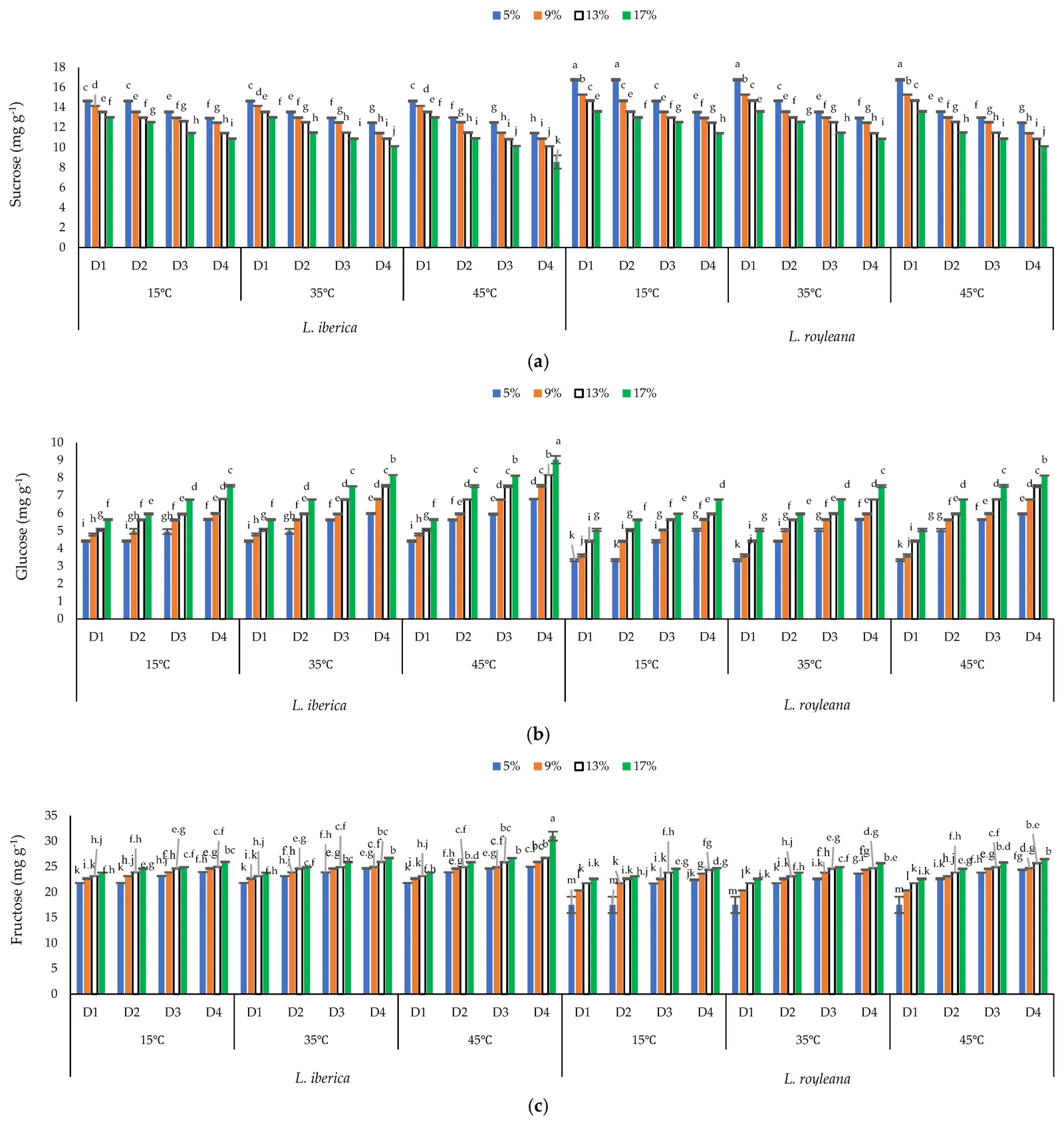
Sucrose (**a**), glucose (**b**), and fructose (**c**) contents in *L. iberica* and *L royleana* seeds under different storage treatments. The bars indicate means ± SEM of three replicates. Different letters indicate significant differences (*p* < 0.05) by Tukey’s multiple range test. For clarity, statistical grouping letters with omitted intermediate letters are presented with a dot (e.g., “a.d” represents “abcd”); the dot has no statistical meaning. Sucrose, glucose, and fructose were determined in seeds immediately after the respective storage treatments. Storage duration (D1: 0, D2: 30 days, D3: 60 days, and D4: 90 days after treatment application).

There was a rise in total soluble sugar (Figure 6b) and sucrose content (Figure 7a) in 5% MC compared to the other moisture levels. A downward trend in total soluble sugar and sucrose content was obtained from 15 °C (cool temperature) to 45 °C (high temperature). Similarly, prolonged storage duration reduced total soluble sugar and sucrose contents. *L. royleana* seeds retained more total soluble sugar and sucrose compared to *L. iberica* (Figure 6b and Figure 7a).

The maximum glucose (Figure 7b) and fructose (Figure 7c) contents were recorded in seeds stored with 17% MC. The greatest increase in these sugars occurred under high-temperature storage (45 °C). Glucose and fructose contents increased significantly from 0 to 90 days. The glucose and fructose contents in *L. iberica* were higher than those in *L. royleana* (Figure 7).

**Figure 8:**
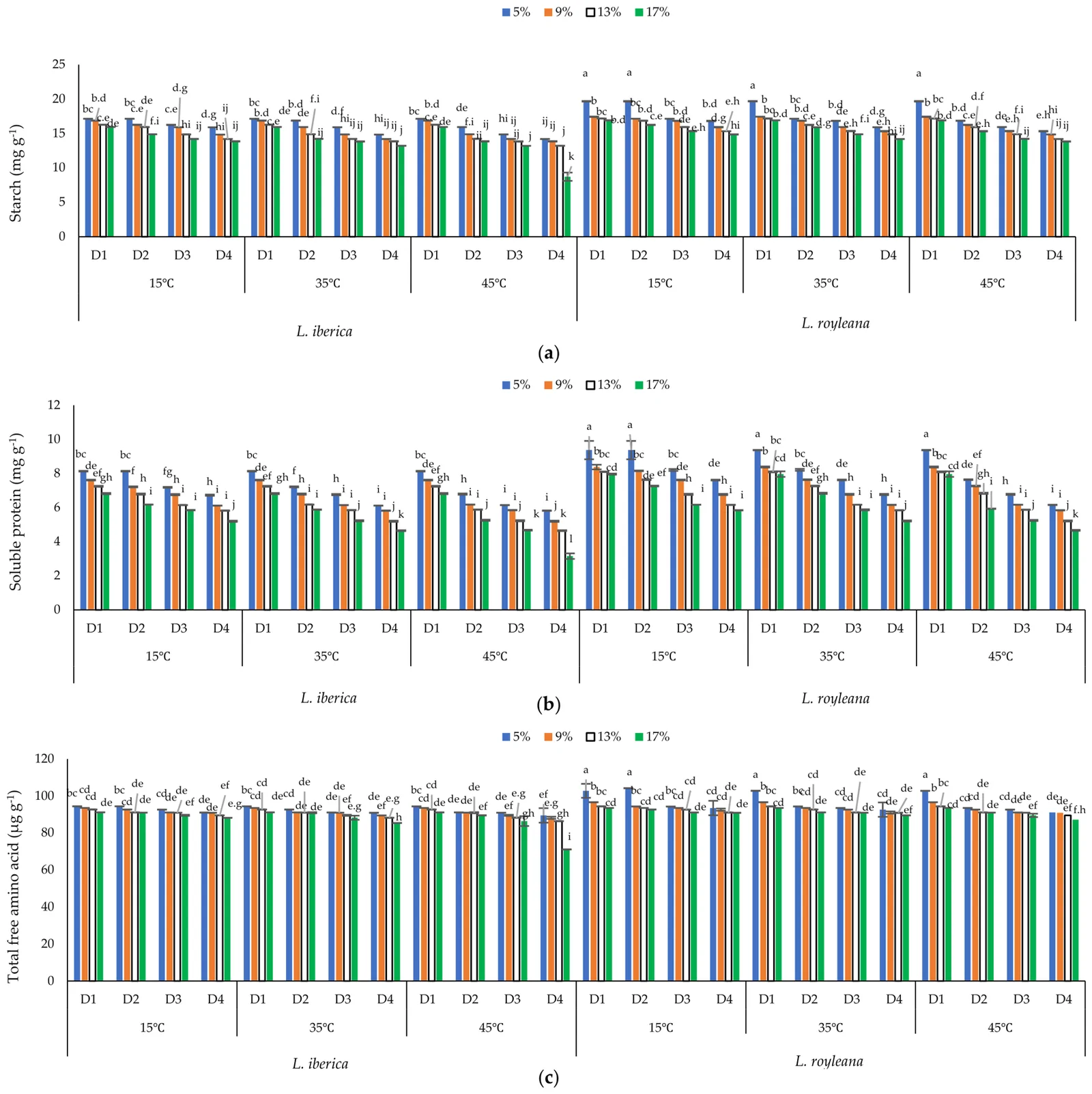
Starch (**a**), soluble protein (**b**), and total free amino acids (**c**) of *Lallemantia* species under different storage treatments. The bars indicate means ± SEM of three replicates. Different letters indicate significant differences (*p* < 0.05) according to Tukey’s multiple range test. For clarity, statistical grouping letters with omitted intermediate letters are presented with a dot (e.g., “a.d” represents “abcd”); the dot has no statistical meaning. Starch, total protein soluble sugar, and total free amino acids were determined in seeds immediately after the respective storage treatments. Storage duration (D1: 0, D2: 30 days, D3: 60 days, and D4: 90 days after treatment application).

The contents of starch (Figure 8a), soluble protein (Figure 8b), and total free amino acids (Figure 8c) declined with increasing seed moisture content. Seed stored at 5% MC preserved the highest starch, soluble protein, and total free amino acids. However, an increase in storage temperature, especially 45 °C (high temperature), accelerated the depletion of starch, soluble protein, and total free amino acids. There was a strong decline in starch, soluble protein, and total free amino acid content in seeds stored from 30 to 90 days. Compared to *L. iberica*, the maximum starch, soluble protein, and total free amino acid contents were recorded in *L. royleana* seeds (Figure 8).

### 2.6. Pearson Correlation

**Figure 9:**
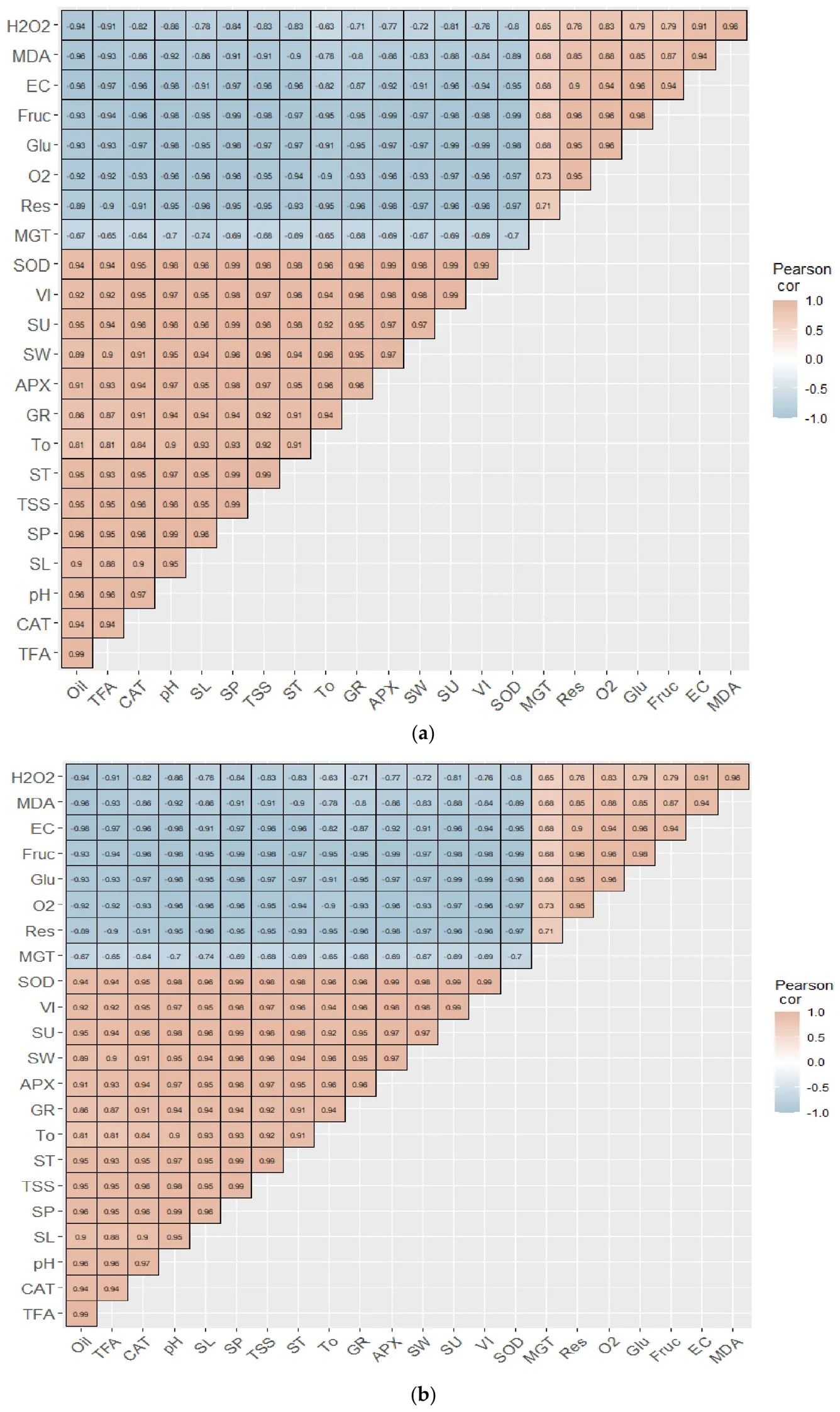
The Pearson correlation data of the studied traits of *L. iberica* (**a**) and *L. royleana* (**b**) under storage conditions. GR: germination rate; MGT: mean germination time; VI: vigor index; SL: seedling length; SW: seedling dry weight; SOD: superoxide dismutase; CAT: catalase; APX: ascorbate peroxidase; To: tocopherol; O_2_•^−^: superoxide anion; H_2_O_2_: hydrogen peroxide; MDA: malondialdehyde; TSS: total soluble sugar; ST: starch; SU: sucrose; Glu: glucose; Fruc: fructose; Res: respiration rate; SP: soluble protein; TFA: total free amino acid.

The Pearson correlation for *Lallemantia* indicated that germination rate showed the strongest correlation with vigor index, seedling growth (seedling length [SL] and seedling dry weight [SW]), antioxidant enzymes (SOD, CAT, and APX), non-enzymatic antioxidant (tocopherol), and seed storage compounds such as soluble protein, sucrose, total soluble sugar, starch, total free amino acid, and oil. Additionally, vigor index showed the most positive correlation with SL, SW, and tocopherol. In contrast, glucose, MGT, O_2_•^−^, EC, respiration rate, fructose, hydrogen peroxide, and MDA exhibited negative correlations with germination rate, VI, seedling growth (SL and SW), antioxidant enzymes (SOD, CAT, and APX) and non-enzymatic antioxidant (tocopherol), seed storage compounds (oil, SP, SU, TSS, ST, TFA) (Figure 9).

### 2.7. Heatmap Analysis

**Figure 10:**
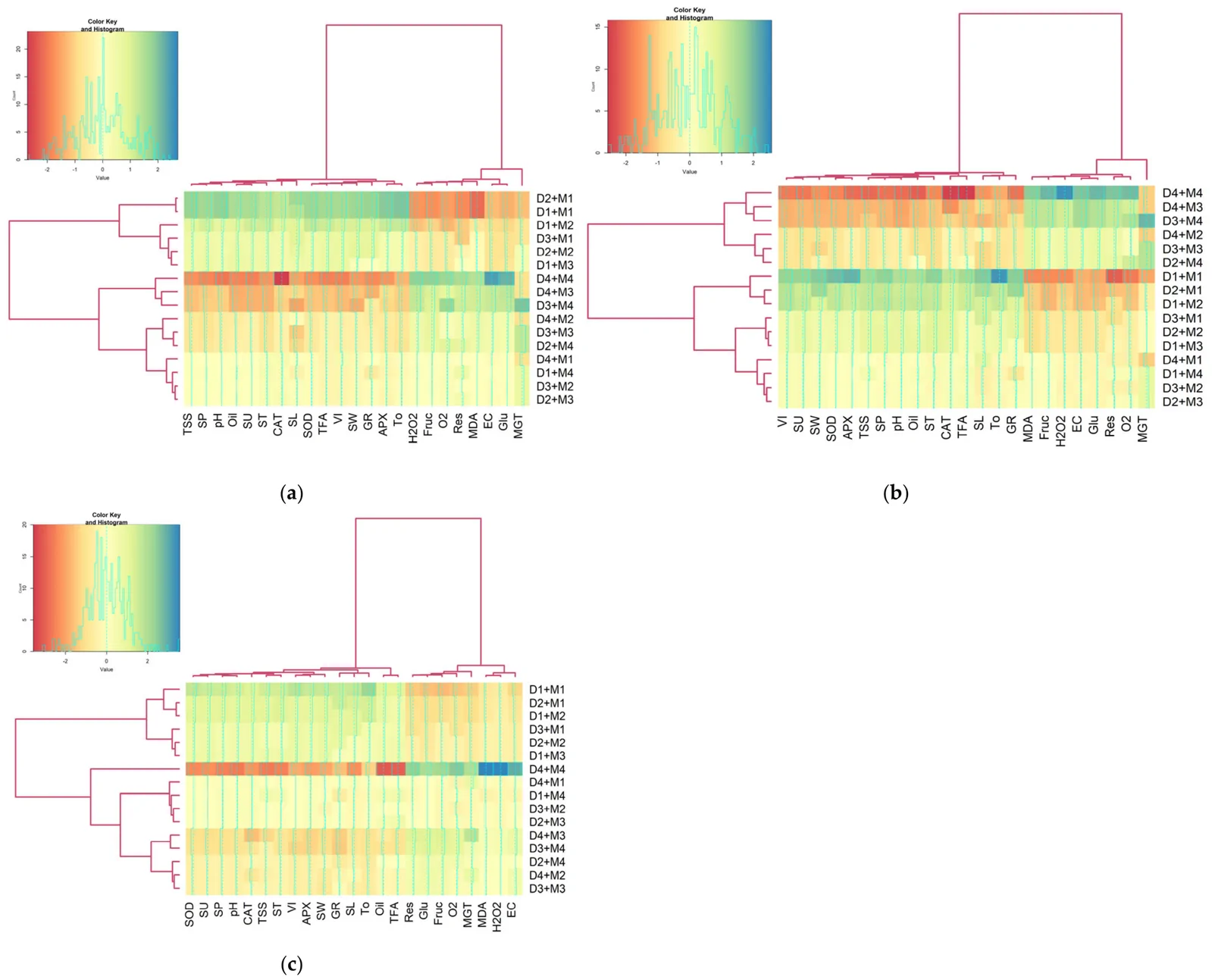
Heatmap analysis indicating the level and type of connection between traits and storage treatments (storage duration and moisture content) in *L. iberica* species at three levels of storage temperature ((**a**): 15 °C; (**b**): 35 °C; (**c**): 45 °C). Each square shows the effect of various treatments on each trait using a false-color scale. Storage treatments included storage duration (D1:0; D2: 30 days; D3: 60 days; D4: 90 days after treatment application) and moisture content (M1: 5%; M2: 9%; M3: 13%; M4: 17%). GR: germination rate; MGT: mean germination time; VI: vigor index; SL: seedling length; SW: seedling dry weight; SOD: superoxide dismutase; CAT: catalase; APX: ascorbate peroxidase; To: tocopherol; O_2_•^−^: superoxide anion; H_2_O_2_: hydrogen peroxide; MDA: malondialdehyde; TSS: total soluble sugar; ST: starch; SU: sucrose; Glu: glucose; Fruc: fructose; Res: respiration rate; SP: soluble protein; TFA: total free amino acid.

**Figure 11:**
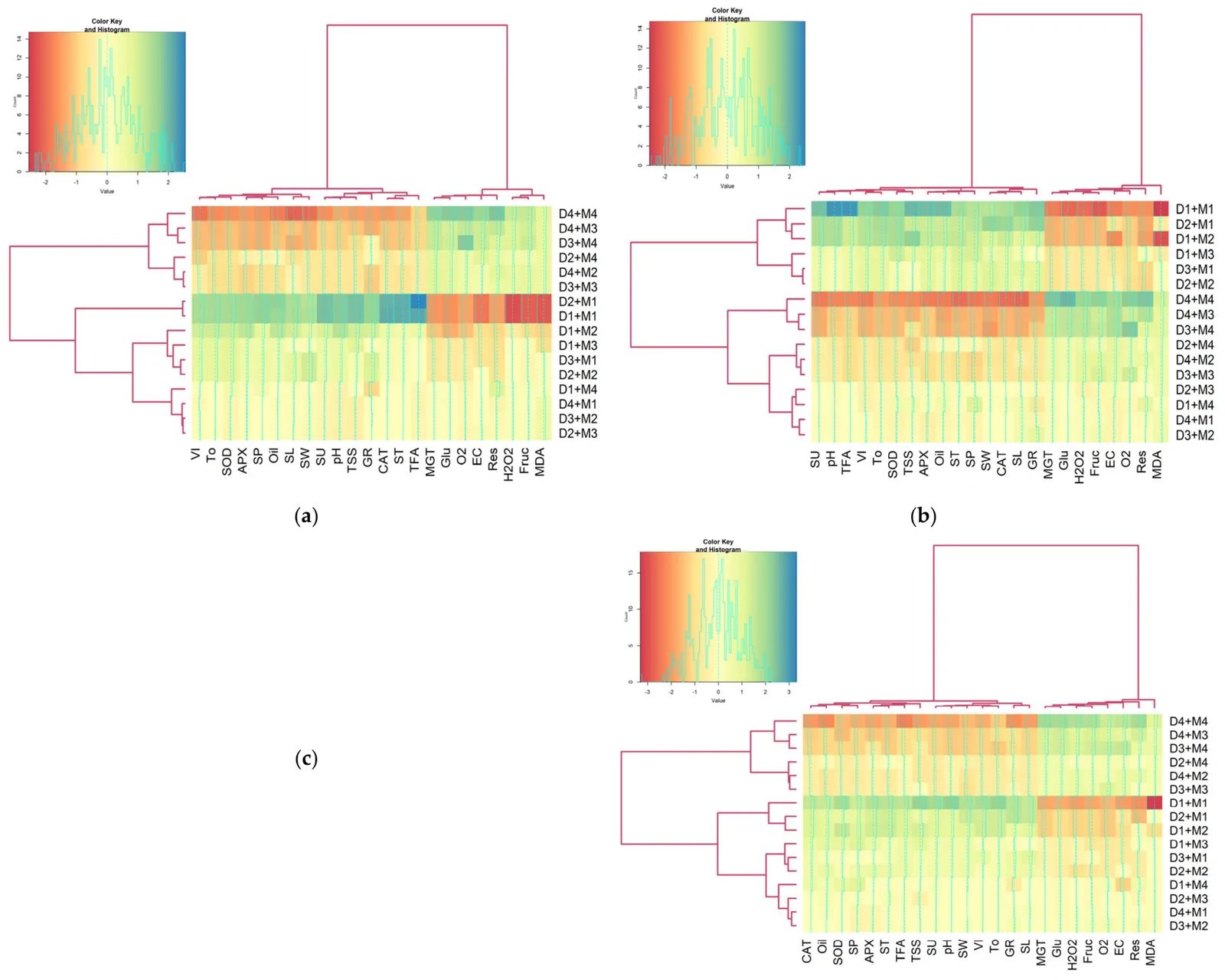
Heatmap analysis indicating the connection between traits and storage treatments (storage duration and moisture content) in *L. royleana* species at three levels of storage temperature ((**a**): 15 °C; (**b**): 35 °C; (**c**): 45 °C). Each square shows the effects of various treatments on each trait using a false-color scale. Storage treatments included storage duration (D1:0; D2: 30 days; D3: 60 days; D4: 90 days after treatment application) and moisture content (M1: 5%; M2: 9%; M3: 13%; M4: 17%). GR: germination rate; MGT: mean germination time; VI: vigor index; SL: seedling length; SW: seedling dry weight; SOD: superoxide dismutase; CAT: catalase; APX: ascorbate peroxidase; To: tocopherol; O_2_•^−^: superoxide anion; H_2_O_2_: hydrogen peroxide; MDA: malondialdehyde; TSS: total soluble sugar; ST: starch; SU: sucrose; Glu: glucose; Fruc: fructose; Res: respiration rate; SP: soluble protein; TFA: total free amino acid.

A correlation heatmap was generated to provide a comprehensive overview of the effects of the storage treatments (moisture content, storage temperature, and storage duration) on germination indices, seedling growth, oxidative products, enzymatic and non-enzymatic antioxidants, and seed storage compounds of *L. iberica* and *L. royleana* (Figure 10 and Figure 11). The heatmap for stored seeds of *L. iberica* at 15 °C (Figure 10a) and 35 °C (Figure 10b) indicated that D2 + M1 and D1 + M1 were the most beneficial treatments in increasing the Gr, SL, SW, VI, enzymatic antioxidants (SOD, CAT, and APX), non-enzymatic antioxidants (tocopherol), and seed storage compounds (TSS, SU, ST, SP, TFA, oil) during storage. In contrast, D4 + M4 at 45 °C had the highest effect on MGT, EC, O_2_•^−^, H_2_O_2_, MDA, glucose, and fructose (Figure 10c). In *L. royleana* seeds, D1 + M1, D2 + M1, and D1 + M2 at 15 °C (Figure 11a) and 35 °C (Figure 11b) were effective for traits such as germination rate, seedling growth, enzymatic and non-enzymatic antioxidants, and seed storage compounds. However, at 45 °C, D4 + M4 further influenced traits such as MGT, oxidative products, glucose, and fructose (Figure 11c).

## 3. Discussion

Seed quality and storage conditions are critical determinants in maintaining seed viability and vigor [38]. However, when seeds have high moisture contents, elevated temperatures, and long storage periods, these conditions accelerate deterioration, thereby reducing seed quality and limiting their agricultural application [39].

Validation of the derived constants (K_E_, C_W_, C_H_, and C_Q_) confirmed that the seed viability equation can be used to describe and predict seed longevity in both *Lallemantia* species. The significantly lower K_E_ value in *L. iberica* than in *L. royleana* indicates a lower estimated inherent seed longevity in *L. iberica*. In addition, the significantly lower C_W_ value in *L. iberica* suggests that the two species respond differently to seed moisture content during storage. However, although C_H_ and C_Q_ were numerically higher in *L. iberica*, the differences between the two species were not statistically significant. Therefore, the observed differences in temperature-related parameters require cautious interpretation and may not be considered evidence of a statistically supported difference in temperature sensitivity between the two species (Table 2). These findings highlight the importance of considering species-specific seed storage characteristics when developing storage protocols. Several crops have shown shelf-life extension when stored under optimal conditions [1,40]. In our experiments, we observed strong shelf-life extension when seeds of both species were stored dry (5% MC) at 15 °C for 30 days (Figure 1a). The lower inherent longevity estimated for *L. iberica* compared with *L. royleana* suggests that maintaining appropriate storage conditions may be particularly important for preserving the viability of *L. iberica*. Consistent with our results, other studies have indicated that seeds stored at higher moisture content and under high temperatures may experience accelerated aging during storage [1]. Even at optimal moisture conditions (5% MC), a decline in seed viability of both *Lallemantia* species under high temperature (45 °C) over 30 days was observed, which may be attributed to temperature-induced membrane deterioration and alterations in the glassy state of the cytoplasm, potentially resulting from enhanced lipid peroxidation [41]. Studies have shown that after storage at low temperatures with lower moisture contents under seed-bank conditions, the cytoplasm remained in a glassy state [8]. In contrast, increased temperature and moisture content influence the glassy state of the cytoplasm and convert it to a fluid state, resulting in accelerated lipid peroxidation and metabolite changes in aged seeds [42,43].

Seed quality and viability are commonly assessed using germination parameters such as germination percentage, germination rate, mean germination time, and vigor index. In the present study, all germination indices declined at the highest MC (17%), storage temperature (45 °C), and storage duration (90 days). A downward trend likely reflects the influence of unfavorable storage conditions [15]. The highest viability was shown in stored seeds at 5% MC and 15 °C for 30 days (Figure 1a), likely because of greater activity of antioxidant enzymes (SOD, CAT, and APX) [44], along with the non-enzymatic antioxidants such as tocopherol [45]. A more pronounced reduction in germination (Figure 3a) in *L. iberica* than in *L. royleana* was accompanied by greater H_2_O_2_ accumulation (Figure 4b), higher O_2_•^−^ levels (Table 4), and greater lipid peroxidation (Figure 4c). This pattern is consistent with previous reports on the membrane-damaging effects of oxidative stress [46] and suggests that the greater loss of germination in *L. iberica* was associated with a stronger oxidative response.

There was a gradual rise in MGT (Figure 3b) with increasing moisture content, storage temperature, and storage duration. This pattern may be explained by the buildup of oxidative compounds and lipid peroxidation during storage [16]. The higher MGT observed in *L. iberica* than in *L. royleana* (Figure 3b) was associated with greater lipid peroxidation and lower storage reserve content. Similar relationships between seed deterioration, oxidative damage, and storage reserves have been reported previously [37].

Length and dry weight of seedlings (Table 3) declined with increasing moisture content, storage temperature, and duration of storage, probably because of the elevated oxidative products [1]. The seed aging process triggers excessive ROS production, causing damage to DNA, RNA, and cellular membranes, and consequently suppressing mitotic activity in meristems [39,47]. The greater seedling length and dry weight (Table 3) observed in *L. royleana* than in *L. iberica* may be associated with higher levels of storage reserves, including starch, sucrose, proteins, free amino acids, and lipids, which can support seedling growth and development [48].

The present study showed that the highest loss of respiratory activity (Figure 4a) in seeds stored at 17% MC, 45 °C, and 90 days may be associated with mitochondrial dysfunction and decreased metabolic efficiency [1]. These circumstances cause electron leakage and accumulation of ROS, including O_2_•^−^ and H_2_O_2_ [11]. The respiration rate was higher in aged *L. iberica* seeds than in aged *L. royleana* seeds (Figure 4a), and *L. iberica* also had lower oil content (Figure 6a). Previous studies have shown that oilseeds rich in polyunsaturated fatty acids are particularly susceptible to lipid peroxidation during storage [49], a process that can contribute to ROS accumulation and the formation of secondary oxidation products [18]. These observations may indicate greater metabolic activity and lipid turnover in aged *L. iberica* seeds [50], although this relationship remains to be tested directly.

The study also showed a consistent rise in the electrical conductivity of seed leachates (Table 4) under all storage treatments, showing a decline in seed vigor and quality [16]. The greatest EC and lowest pH (Table 4) at 17% MC, 45 °C, and 90 days reflected extensive membrane damage. As seed deterioration progresses, cell membranes lose integrity, increasing the leakage of sugars, organic acids, and hydrogen ions (H^+^) [49]. Also, the accumulation of acidic products lowers the pH of the leachate [50]. In this study, the higher electrical conductivity and leachate pH observed in *L. iberica* than in *L. royleana* (Table 4) coincided with evidence of greater oxidative products such as H_2_O_2_ (Figure 4b), O_2_•^−^ (Table 4), and MDA (Figure 4c) during seed deterioration. These differences may be related to changes in membrane integrity and metabolic activity associated with deterioration [10].

The highest H_2_O_2_ (Figure 4b), O_2_•^−^ (Table 4), and MDA (Figure 4c) contents were detected in seeds stored under 17% MC and 45 °C for 90 days, matching previous reports [51,52]. Elevated MDA content detected in aged seeds indicates greater lipid peroxidation and damage to the plasma membranes. In this study, *L. iberica* seeds exhibited higher H_2_O_2_ (Figure 4b) and O_2_•^−^ (Table 4) levels, as well as greater TBA content (Figure 4c), than *L. royleana* seeds. These differences coincided with lower oil content (Figure 6a) and lower relative percentages of unsaturated fatty acids, namely LNA, LA, and OA, together with higher percentages of palmitic and stearic acids (Table 6). Because unsaturated fatty acids are the main substrates of lipid peroxidation [53,54], this pattern may be consistent with greater oxidative loss of unsaturated lipids in *L. iberica*. However, the present results do not establish a causal link, and without data on the initial fatty-acid composition, an inherent compositional difference between the two species cannot be excluded. Other TBA-reactive compounds, including soluble sugars and their degradation products, may interfere with spectrophotometric measurements, particularly under prolonged or severe storage conditions [55]. In the absence of HPLC confirmation, these results are therefore interpreted as changes in TBARS rather than as a definitive quantification of MDA.

Antioxidant and non-antioxidant defense systems play an essential role in regulating ROS balance and maintaining cellular homeostasis [15,56]. The lowest activities of SOD (Figure 5a), CAT, APX (Table 4), and tocopherol (Figure 5b) were observed in seeds stored under 17% MC, 45 °C for 90 days, which is probably owing to the accumulation of oxidative products [46]. Tocopherol increases antioxidant defense by stimulating enzymes such as SOD, CAT, and APX, which scavenge ROS and prevent membrane degradation [49,57,58]. There was greater antioxidant enzyme activity in *L. royleana* than in *L. iberica,* and this coincided with lower contents of H_2_O_2_ (Figure 4b), O_2_•^−^ (Table 4) and MDA (Figure 4c). This pattern is consistent with the role of antioxidant enzymes in limiting ROS accumulation and oxidative damage [38,59].

Furthermore, storage of *Lallemantia* seeds at 45 °C with a 17% MC for 90 days resulted in changes in the fatty acid profile, with decreases in the relative percentages of unsaturated fatty acids such as LNA, LA, and OA (Table 6), accompanied by changes in the relative proportions of saturated fatty acids such as stearic acid and palmitic acid. The greater decline in unsaturated fatty acids may be associated with their higher susceptibility to oxidative degradation due to the presence of double bonds [60]. Oxidation of seed reserves is a major biochemical marker of deterioration [8] and is often accompanied by membrane disruption, which may facilitate oxygen diffusion and promote lipid peroxidation and lipid degradation [61]. Reduced enzymatic and non-enzymatic antioxidant activities during storage have been associated with increased lipid peroxidation, reduced oil content, and alterations in fatty acid profiles [15,62]. *L. iberica* showed lower oil content (Figure 6a) and lower relative percentages of the unsaturated fatty acids LNA, LA, and OA, together with higher percentages of palmitic and stearic acids (Table 6), than *L. royleana* under the storage conditions examined. Together with its higher H_2_O_2_ (Figure 4b), O_2_•^−^ (Table 4) and MDA (Figure 4c), this pattern may be consistent with greater lipid deterioration in *L. iberica* [63].

The higher levels of total soluble sugars (Figure 6b), sucrose (Figure 7a), and starch (Figure 8a) under 5% MC and 15 °C therefore likely reflect better preservation of pre-existing carbohydrate reserves, rather than active carbohydrate metabolism during dry storage [1]. In addition, lower storage temperatures limit oxidative deterioration and the accumulation of reactive oxygen species, thereby contributing to the preservation of carbohydrate reserves during storage [64,65]. Consequently, optimal storage conditions minimize the physicochemical degradation of reserve carbohydrates and maintain higher levels of total soluble sugars, sucrose, and starch [1,66,67]. Total soluble sugars (Figure 6b), sucrose (Figure 7a), and starch (Figure 8a) contents were lower in *L. iberica* than in *L. royleana*. This pattern coincided with the lower antioxidant enzyme activities and higher H_2_O_2_ (Figure 4b), O_2_•^−^ (Table 4) and MDA (Figure 4c) contents, as well as the higher respiration rate (Figure 4a) observed in *L. iberica*. Oxidative stress and the metabolic consumption of reserves during aging have both been associated with carbohydrate depletion in stored seeds, and loss of stability of the cytoplasmic glassy state has been proposed as a further contributor to deterioration [41,43,68].

The content of glucose (Figure 7b) and fructose (Figure 7c) increased with increasing seed moisture content, storage temperature, and storage duration. These increases may be associated with the greater physicochemical and oxidative deterioration occurring under more severe storage conditions [69]. Reactive oxygen species accumulated during storage can contribute to oxidative damage of membranes and storage macromolecules, potentially promoting slow physicochemical modification and fragmentation of carbohydrate reserves [13,17]. Therefore, the increased glucose and fructose contents observed in aged seeds are more plausibly associated with slow non-enzymatic physicochemical deterioration, and possibly limited chemical or residual enzymatic reactions at higher moisture contents [15,35]. At the higher moisture contents used in this study, particularly 17% MC, increased molecular mobility may have permitted limited chemical and possibly residual enzymatic reactions; however, these may not count as active metabolism that characterizes fully imbibed seeds. In contrast to sucrose (Figure 7a) and starch (Figure 8a), glucose (Figure 7b) and fructose (Figure 7c) contents were higher in aged *L. iberica* seeds under severe storage conditions. This pattern, together with the lower sucrose and starch contents, is consistent with the hydrolysis of storage carbohydrates during deterioration [5], although it may also be influenced by the higher respiration rate and oxidative stress observed in this species. Because glucose and fructose constituted only a small fraction of the total soluble sugar pool (Figure 6b) [70], the decrease in sucrose, the predominant soluble sugar, exceeded the increase in these monosaccharides, resulting in lower total soluble sugars in *L. iberica* (Figure 6b). It should also be noted that elevated glucose and fructose may contribute to the higher TBARS values in this species, as reducing sugars can interfere with the TBA assay [71].

A reduction in soluble protein (Figure 8b) and total free amino acid (Figure 8c) content was observed under 17% MC and 45 °C for 90 days of storage. This reduction is more likely attributable to oxidative modification, aggregation, and cross-linking of storage proteins, along with physicochemical degradation and, particularly at higher moisture content, possible limited proteolytic activity during storage, rather than reduced protein synthesis, which does not occur in the dry seed state [72,73]. Soluble protein (Figure 8b) and total free amino acid (Figure 8c) contents were lower in *L. iberica* than in *L. royleana*, which coincided with its higher H_2_O_2_ (Figure 4b) and O_2_•^−^ (Table 4) and MDA (Figure 4c) contents. Lipid peroxidation is known to generate ROS and reactive aldehydes such as MDA, which can modify amino acids and proteins through oxidation, carbonylation, and cross-linking [11,18,59,74].

In this study, we observed negative correlations between EC, pH, H_2_O_2_, O_2_•^−^, MDA, glucose, and fructose and key markers of physiological quality, such as germination rate, viability, antioxidant system, and seed storage compounds, in both species (Figure 9a,b). The negative correlations further indicate a relationship between biochemical modifications during storage and the degradation of seed physiological quality. Negative correlations between the physicochemical parameters and reduced germination rate and viability may reflect changes in membrane integrity. Similarly, the negative relationship between the accumulation of ROS and germination rate and viability is consistent with increased oxidative stress linked to deterioration of cellular components during storage [16,75]. Soluble sugars such as glucose and fructose play crucial roles in seed metabolism and stress responses, and changes in their levels can be associated with changes in seed physiological quality [25,26,76]. The negative associations observed between these sugars and germination rate and viability may indicate non-enzymatic alterations in carbohydrate metabolism during storage that accompany the loss of seed quality [26]. Seed deterioration in storage is characterized by metabolic changes, including changes in soluble sugar levels [25]. Moreover, at a relatively high MC of 17%, the increase in glucose and fructose may also suggest changes in carbohydrate breakdown, although the specific mechanism underlying these changes in the present study is uncertain.

The correlation heatmaps revealed associations between storage treatments and seed physiological and biochemical traits in both *L. iberica* and *L. royleana* (Figure 10 and Figure 11). In both species, treatments involving lower seed moisture content and storage temperature, particularly D1 + M1 and D2 + M1, were associated with higher germination, greater seedling growth, higher antioxidant activity, and better maintenance of seed storage compounds. In contrast, the D4 + M4 treatment was associated with higher mean germination time, elevated oxidative stress markers, and increased soluble sugar levels. Overall, these patterns suggest that lower storage temperature and seed moisture content were associated with better maintenance of seed physiological and biochemical quality over the 90-day experimental period, whereas higher temperature and moisture content coincided with more pronounced oxidative and metabolic changes. These observations are consistent with previous studies reporting accelerated seed deterioration under high-temperature and high-moisture conditions [26,34]. However, the correlations observed in this study do not establish causal relationships between storage conditions and individual physiological or biochemical responses, nor do they directly predict seed longevity under conventional long-term storage conditions.

## 4. Materials and Methods

### 4.1. Experimental Design and Storage Treatments

This study was performed as a factorial experiment based on a completely randomized design with three replications. The factors for this storage condition study included storage temperature (15 °C, 35 °C, and 45 °C), duration (0, 30, 60, and 90 days), seed moisture content (5, 9, 13, and 17%), and plant species (*L. iberica* and *L. royleana*). The temperatures were selected to impose distinct environmental pressures (from a level at which metabolic and deteriorative processes are slowed down to levels at which aging is substantially accelerated). The selected storage temperatures represented contrasting storage environments (cool (15 °C), warm (35 °C), and high (45 °C) temperature conditions). Together with different seed moisture contents and storage durations, these factors were evaluated to determine their individual and interactive effects on seed deterioration. The study evaluated variations in the measured parameters among treatments over the 90-day observation period. Initial measurements were taken after applying the respective treatments and served as the starting point for subsequent measurements at 30, 60, and 90 days of storage.

### 4.2. Seed Sources

*Lallemantia iberica* and *L. royleana* seeds were collected from the research farm in Minab, Hormozgan (57°05′13.8 E, 27°07′51.7 N, altitude: 43 m above sea level) in December 2024. The initial moisture content was 5.86% in *L. iberica* and 7% in L. *royleana* (Table 7), determined by a moisture meter (DBS 60-3, KERN & SOHN GmbH, Balingen-Frommern, Germany). To obtain the target moisture content of 5%, the seeds were gradually dried under controlled conditions until the desired moisture level was reached. The moisture content was subsequently verified using the same moisture meter before initiating the storage treatments.

### 4.3. Moisture Content (MC)

Seed moisture content was measured by drying three replicates of 5 g of seeds in an oven at 103 °C for 17 h, followed by reweighing. The initial moisture content was approximately 5.86% for *L. iberica* and 7% for *L. royleana*. To achieve higher moisture levels (9%, 13% and 17%), a calculated amount of distilled water was added to the seeds in aluminum foil bags according to Equation (1) [77].

W_2_ = ((A − B)/100 − A) × W_1_ (1)

In Equation (1), A represents the target moisture content, B is the initial moisture content (%), W_1_ is the initial weight of seed mass (g), and W_2_ is the mass of water to add. Approximately 18 g of seeds at each target MC (5, 9, 13, and 17%) were sealed in aluminum foil bags and held at 10 °C for 24 h to reach equilibrium and achieve uniform moisture distribution while preventing the initiation of pre-germination metabolic activity. The airtight containment ensured even moisture distribution across the seed batch through vapor-phase and matrix equilibration. The seeds were not surface-sterilized either before or after storage. Throughout the storage period, no visible fungal growth, pest infestation, or other signs of microbial contamination were observed in any treatment, including the 9, 13, and 17% MC treatments, and the seeds remained visually healthy. Following equilibration, three bags, each containing at least 400 seeds, were used to re-measure the seed moisture content and verify that the target MC had been reached. The remaining sealed seed samples were placed in incubators (Heraeus, Thermo Fisher Scientific Inc., Hanau, Germany) at 15, 35, and 45 °C. Samples were collected every 30 days for up to 90 days.

### 4.4. Measurements

#### 4.4.1. Modelling Seed Viability Loss and Determination of C_W_, K_E_, C_H_, and C_Q_ (Viability Constants)

The model for seed longevity, or the decline in viability over time under storage conditions, was applied using the following equations [78,79], describing the linear decline in seed viability over storage time.

V = Ki − p/*σ* (2)

The viability (*V*) is expressed in probits, a statistical unit that normalizes the sigmoidal survival curve. In this model, *Ki* is the initial seed lot quality, representing the intercept as the probit viability at time zero, and *P* is the storage duration (days). The parameter *σ* defines the time (in days) for viability to decrease by one probit (one Normal Equivalent Deviation), which is the slope of the line and a direct measure of seed longevity. A larger σ indicates a slower loss of viability and thus greater seed longevity.

Log_10_σ = K_E_ − C_w_log_10_m − C_H_t − C_Q_t^2^ (3)

σ = 10^(KE−Cwlog10m−CH−CQt2)^ (4)

This equation quantifies how the storage environment, specifically moisture content (*m*) and temperature (*t*), influences the seed longevity parameter (*σ*). The constants *K_E_* and *C_W_* are species-specific. *K_E_* represents the theoretical inherent longevity (log *σ*) at a baseline of 1% moisture content and 0 °C, while C_W_ quantifies the sensitivity of longevity to changes in moisture content. In contrast, the temperature coefficients *C_H_* and *C_Q_* are universal for all orthodox seeds and describe a consistent quadratic relationship between temperature and log *σ*.

#### 4.4.2. Germination Indices and Seedling Vigor Index

Germination was determined with three replications of 20 seeds each. Seeds were placed in 9 cm Petri dishes, each receiving 10 mL of water. The amount of water was sufficient to moisten the germination substrate without submerging the seeds, which remained exposed to air throughout the germination test. The germination test was performed over 14 days under a 16/8 h fluorescent-white light/dark cycle at 10 °C and 85% relative humidity [37]. A seed was scored as germinated once it had radicle protrusion of at least 2 mm. Germination rate (GR), mean germination time (MGT), seedling vigor index (SVI), seedling length (SL), and seedling dry weight (SW) were calculated according to the following equations [38]:

(5) $$\mathrm{GR}\;=\;\frac{1}{MGT}$$

(6) $$\mathrm{MGT}=\frac{\Sigma niti}{\Sigma ni}$$

In Equation (5), MGT represents the mean germination time. In Equation (6), *n_i_* represents the number of seeds germinated at time *t_i_*, and *t_i_* represents the time (days) from the beginning of the germination test to the *i*th observation.

After germination, seedling length and dry weight were determined at the end of the germination test (14 days), conducted at 10 °C under a 16/8 h fluorescent-white light/dark cycle and 85% relative humidity. Seedling dry weight (SW) was calculated after drying the seedlings at 70 °C for 24 h. Their lengths were manually measured using a ruler in mm, and the mean seedling length (SL) was calculated. For each replicate, 10 germinated seedlings were randomly selected for the assessment of seedling traits. The seedling vigor index (SVI) was measured using Equation (7).

SVI = GP (Germination percentage) × SL(7)

#### 4.4.3. Respiration Rate

Respiratory rate was measured with an infrared gas analyzer (GHX-3010E1, Beijing Huayun Analysis Equipment Institute, Beijing, China). Approximately 1 g of hydrated, germinated aged seeds was placed in a 200.96 mL air chamber, and CO_2_ levels were calculated every 15 s. After a 1-min stabilization period, nine readings were collected over 120 s. The respiratory rate was calculated by the difference between the initial and final CO_2_ concentrations (ppm) and expressed in μmol CO_2_ g^−1^ min^−1^ [80].

#### 4.4.4. Electrical Conductivity and pH of Leachate

To measure the electrical conductivity and pH of the leachate, 100 aged seeds per replicate were placed in 50 mL of deionized water and incubated at 20 °C for 24 h, with three replicates in total [77]. Then, an EC meter (Thermo Scientific, Waltham, MA, USA, Orion Star A212 EC Meter) and a pH meter (Thermo Scientific, Orion Star A211 pH Meter) were used to measure the electrolyte solution.

#### 4.4.5. Oxidative Products

Hydrogen peroxide (H_2_O_2_) content was determined according to Loreto and Velikova [81]. Briefly, 200 mg of aged seeds was extracted with 5 mL of 0.1% (*w*/*v*) trichloroacetic acid (TCA, ≥99.5%, Merck KGaA, Darmstadt, Germany) in an ice bath. After centrifugation at 13,000× *g* for 20 min, 0.2 mL of 10 mM phosphate buffer (pH 7.0) was combined with 1 mL of 1 M potassium iodide (≥99.0%, Merck KGaA, Darmstadt, Germany). After 1 h of incubation, absorbance was measured at 390 nm using a spectrophotometer (U-2000, Hitachi Instruments, Tokyo, Japan). H_2_O_2_ concentration was determined using a standard calibration curve prepared with known H_2_O_2_ concentrations ranging from 2 to 10 mM, following the procedure described by Loreto and Velikova [80]. The results were expressed as nmol g^−1^ dry weight.

Superoxide (O_2_^−^•) content was determined as described by Jiang and Zhang [82]. About 100 mg of aged seeds was homogenized in 3 mL of 65 mM potassium phosphate buffer (pH 7.8), followed by centrifugation at 5000× *g* for 10 min. For the assay, 1 mL of the resulting supernatant was combined with 0.9 mL of 65 mM phosphate buffer (pH 7.8) and 0.1 mL of 10 mM hydroxylamine hydrochloride. The mixture was incubated at 25 °C for 1 h. Subsequently, 17 mM sulfanilamide and 7 mM α-naphthylamine were added, and the reaction was allowed to proceed at 25 °C for an additional 20 min. An equal volume of ethyl ether was then added, and the mixture was centrifuged at 1500× *g* for 5 min. The absorbance of the aqueous phase was measured at 530 nm using the same spectrophotometer (U-2000, Hitachi Instruments, Tokyo, Japan). Sodium nitrite (NaNO_2_) standard solutions were prepared at concentrations of 0, 10, 20, 30, 40, and 50 µM to construct the calibration curve. The O_2_^−^• content in the samples was calculated using this calibration curve and expressed as µmol g^−1^ dry weight.

Lipid peroxidation was assessed by measuring malondialdehyde (MDA) according to Heath and Packer [83]. Approximately 200 mg of aged seeds was homogenized in 5 mL of 0.5% (*w*/*v*) thiobarbituric acid (TBA, ≥98.0%, Merck KGaA, Germany) and centrifuged at 15,000× *g* for 20 min. Subsequently, 1.0 mL of the supernatant was combined with 2.0 mL of 0.5% TBA, heated at 100 °C for 15 min, and cooled in an ice bath. Absorbance was measured at 532 and 600 nm using the U-2000 spectrophotometer (Hitachi Instruments, Tokyo, Japan). MDA concentration was calculated from the difference between A_532_ and A_600_ using a molar extinction coefficient of 155 mM^−1^ cm^−1^ and expressed as nmol g^−1^ dry weight.

#### 4.4.6. Enzymatic and Non-Enzymatic Antioxidant Assays

The activity of superoxide dismutase (SOD) was calculated using 200 mg of aged seeds, which were homogenized in 2 mL of 50 mmol L^–1^ phosphate buffer (pH 7.8) and centrifuged in 10 mL tubes. The resulting supernatant was used as the enzyme extract. The reaction mixture contained 12 mM methionine (Met), 2 mM EDTA-Na_2_ (≥99.0%, Merck KGaA, Germany), 3.25 mM nitroblue tetrazolium (≥90.0% NBT, Sigma-Aldrich, St. Louis, MO, USA), and 50 μM riboflavin (≥98.0%, Sigma Aldrich, USA). Assays were performed by adding 2.8 mL of the reaction mixture and 0.2 mL of the enzyme extract to the tube, while the control received 0.1 mL of phosphate-buffered saline (PBS) instead of the enzyme extract. The reaction tubes were exposed to light at 25 °C for 20 min, and absorbance was measured at 560 nm with a spectrophotometer (U-2000, Hitachi Instruments, Tokyo, Japan) [84]. Catalase enzyme activity (CAT) was measured using the enzyme extract prepared for SOD determination. The reaction mixture contained 50 mL of 0.15 mol L^–1^ phosphate buffer (pH 7.0) and 0.05 mL of 30% H_2_O_2_ (Merck KGaA, Germany). For each assay, 2.8 mL of the reaction mixture was combined with 0.1 mL of enzyme extract, and absorbance was recorded at 240 nm for 30 s [85]. Ascorbate peroxidase (APX; EC 1.11.1.11) activity was determined using the same enzyme extract prepared for the SOD assay. The reaction mixture contained 2.6 mL of 0.05 mol L^−1^ phosphate buffer (pH 7.0) supplemented with 0.1 mM EDTA-Na_2_, 0.15 mL of 5 mM ascorbic acid (≥99.0%, Sigma-Aldrich, USA), and 0.10 mL of enzyme extract. The reaction was initiated by adding 0.15 mL of 20 mM H_2_O_2_, and the decrease in absorbance was monitored at 290 nm at 30-s intervals [86].

To measure tocopherol content (vitamin E), briefly, 5 g of aged seeds was extracted with 10 mL of acetone:methanol (7:3) (≥99.9%, Merck KGaA, Darmstadt, Germany)) and centrifuged at 11,000× *g* for 30 min. The assay mixture was prepared by combining 1 mL 0.5% 2-2 bipyridine (≥99.0%-Sigma Aldrich, USA), 1 mL 0.2% iron chloride (≥99.0%-Merck KGaA-Germany), and 1 mL supernatant. Absorbance was calculated at 522 nm after 60 s, and α-tocopherol was used for calibration [87].

#### 4.4.7. Metabolite Analysis

Approximately 1 g of ground seeds was placed in a Soxhlet extraction thimble. Hexane (150 mL; ≥96.0%, Merck KGaA, Germany) was used as the extraction solvent, and extraction was performed at 40–60 °C for 4 h. After extraction, the solvent was removed by evaporation, and the extracted oil was recovered and filtered prior to further analysis [88]. Fatty acid composition was determined by gas chromatography (GC) following methylation to fatty acid methyl esters (FAMEs), according to Savage et al. [89]. Briefly, 0.2 g of seed oil was mixed with 2 mL of heptane and 300 μL of methanolic potassium hydroxide and centrifuged at 11,000× *g* for 10 min at 4 °C. The resulting FAME fraction was collected, and 3 μL was injected into the GC system using a microsyringe. Fatty acids were separated on a BPX70 capillary column (Agilent Technologies, Inc., Santa Clara, CA, USA). The oven temperature was initially maintained at 165 °C for 10 min and then increased to 200 °C at a rate of 15 °C min^−1^. The injector and detector temperatures were maintained at 250 and 280 °C, respectively. Instead, a standard fatty acid mixture at concentrations of 2, 5, 10, 25, and 50 mg mL^−1^ was subjected to the same methylation procedure and used for fatty acid quantification. The total soluble sugars were extracted from 0.1 g of aged seeds with 10 mL of 80% ethanol (≥99.9%, Merck KGaA, Darmstadt, Germany). The sample tubes were placed in an ice bath, and 10 mL of anthrone reagent was slowly added to each tube. The tubes were then incubated in a boiling water bath for exactly 7.5 min and immediately cooled in an ice bath. After cooling, the absorbance was measured at 620 nm within 1 h [90].

Sucrose, glucose, and fructose were extracted with 80% ethanol at 70 °C in a two-phase extraction procedure [91]. After evaporation, the extracts were drafted to trimethylsilyl ethers with a solution reagent containing pyridine (≥99.5%-Merck KGaA-Germany), hexamethyldisilazane (≥99.0%-Sigma Aldrich, USA), and trimethylchlorosilane (≥99.0%-Sigma Aldrich, USA). Quantification and identification were performed on an HP 5890 Series II instrument. Injector and detector temperatures were set at 280 °C and 300 °C, respectively. The oven program started at 80 °C and increased to 315 °C. Sugars were identified by comparing retention times with those of authentic trimethylsilyl carbohydrate standards.

For starch determination, 0.2 g of aged seeds was homogenized in 2 mL of 80% ethanol. The extract was incubated in a water bath at 30 °C for 30 min and centrifuged at 12,000× *g* for 15 min. The supernatant was discarded, and the pellet was washed with 2 mL of 80% ethanol. The remaining residue was evaporated and resuspended in 2 mL of deionized water, followed by heating at 100 °C for 20 min. Starch hydrolysis was done with 9.2 M and 4.6 M HClO_4_ (Merck KGaA-Germany), and starch content was measured using the anthrone reagent at 480 nm [92].

The soluble protein content was calculated using the same enzyme extract used for SOD determination. For the reaction, about 1 mL of the extract was added to 5 mL of Coomassie Brilliant Blue (Merck KGaA-Germany) solution and allowed to react for 2 min. Absorbance was then measured at 595 nm. Protein content was calculated from a standard curve prepared with 0–100 μg bovine serum albumin [93].

Extraction of total free amino acids was performed by homogenizing 1 g of sample in 10 mL of 80% methanol, incubating at −20 °C for 1 h, and centrifuging at 12,000× *g* for 20 min at 4 °C. The pellet was re-extracted twice, and the supernatants were pooled. Total free amino acids were quantified by the ninhydrin method. For the assay, 1.2 mL of glycerol, 0.2 mL of 0.5 M citrate buffer, 0.5 mL of 1% ninhydrin (≥99.0%, Sigma Aldrich, USA), and 0.1 mL of the methanolic extract were combined. After boiling in a water bath for 12 min and subsequent cooling in an ice bath, the samples’ absorbance was measured at 570 nm. The amino acid content was then determined using a valine calibration curve [94].

#### 4.4.8. Statistical Analysis

The data were analyzed using SAS (version 9.2, SAS Institute, Cary, NC, USA). Data normality and homogeneity of variance were assessed before performing analysis of variance (ANOVA). ANOVA was performed separately for each response variable based on a four-factor factorial model within a completely randomized design with three replications. The fixed factors were storage duration (D; 0, 30, 60, and 90 days), seed moisture content (M; 5, 9, 13, and 17%), storage temperature (T; 15, 35, and 45 °C), and plant species (S; *L. iberica* and *L. royleana*). The ANOVA model included the main effects of D, M, T, and S; their two-way interactions (D × M, D × T, D × S, M × T, M × S, and T × S); three-way interactions (D × M × T, D × M × S, D × T × S and M × T × S); and the four-way interaction (D × M × T × S). Mean comparisons were performed using Tukey’s multiple range test at the 5% significance level. LogWorth values were calculated as −log10(P) from the corresponding ANOVA *p*-values using JMP Pro 16 (JMP Version 16, SAS Institute Inc., Cary, NC, USA). LogWorth values were used to summarize the statistical significance of the tested factors and their interactions and were not interpreted as measures of effect size. Detailed ANOVA and LogWorth results are presented in Supplementary Tables S1–S8. Heatmaps and Pearson correlation analyses were performed using the R packages (version 4.3.1) ‘FactoMineR’ and ‘factoextra’ [95].

## 5. Conclusions

Seeds are essential units of plant propagation, and their value for planting and seed production largely depends on their quality. Our findings showed that, over the 90-day storage period, storage conditions significantly affected seed viability, germination, vigor, seedling growth, antioxidant enzymes (SOD, CAT, and APX), the non-enzymatic antioxidant tocopherol, and major storage compounds, including oil, fatty acids (LNA, LA, OA, PA, and SA), carbohydrate reserves (sucrose, starch, glucose, and fructose), soluble protein, and total free amino acids. Increasing seed moisture content, storage temperature, and storage duration was generally associated with reductions in seed viability, antioxidant capacity, and storage reserves. These changes coincided with increases in electrical conductivity, leachate pH, H_2_O_2_, O_2_•^−^, MDA-related measurements, glucose, and fructose, consistent with membrane deterioration and changes in cellular metabolism during storage. These results indicate that *Lallemantia* seeds are sensitive to unfavorable storage conditions within the tested range, underscoring the importance of appropriate storage management for maintaining seed quality. Among the conditions evaluated, seeds stored at 5% moisture content and 15 °C (cool temperature) generally showed better maintenance of physiological and biochemical quality over the 90 days.

Overall, *L. iberica* showed greater deterioration than *L. royleana* in several measured traits, including greater reductions in viability and antioxidant enzyme activities and greater increases in oxidative stress indicators under the tested storage conditions. The viability equation, fitted to data collected over the 90-day experimental period, yielded a significantly lower model-estimated value of inherent seed longevity (K_e_) for *L. iberica* than for *L. royleana*. This result indicates a difference in model-estimated inherent longevity between the species but does not constitute a direct measurement of their long-term storage life. In contrast, although the temperature-related parameters C_H_ and C_Q_ were numerically higher in *L. iberica*, the differences between species were not statistically significant; therefore, these parameters do not provide sufficient evidence of a species difference in temperature sensitivity. The findings offer preliminary guidance for refining storage strategies for *Lallemantia* seeds. However, because the experiment was limited to 90 days, the results provide only indirect evidence regarding long-term storage performance under practical conditions. Longer-term studies under practical storage conditions are needed to validate these findings before the proposed storage conditions can be recommended for commercial storage or gene bank conservation. Future studies should extend storage duration under practical storage conditions and investigate the molecular and biochemical mechanisms that may underlie the observed interspecific differences in seed deterioration.

## Figures and Tables

**Table 1:** LogWorth values indicating the statistical significance of storage factors and their interactions for germination of *L. iberica* and *L. royleana*.

Source	LogWorth	*p*-Value
Storage duration (D)	102.73	<0.000001
Storage temperature (T)	69.57	<0.000001
Moisture content (M)	63.86	<0.000001
(D) × (T)	30.54	<0.000001
(D) × (M)	29.15	<0.000001
Plant species (S)	29.05	<0.000001
(M) × (T)	21.65	<0.000001
(T) × (S)	8.14	<0.000001
(D) × (M) × (T)	7.41	<0.000001
(D) × (S)	5.55	<0.000001
(M) × (S)	4.67	<0.00002
(D) ×(M) × (T) × (S)	1.93	0.01174
(D) × (M) × (S)	1.43	0.03712
(D) × (T) × (S)	1.03	0.09199
(M) × (T) × (S)	0.35	0.44573

LogWorth = −log10(P). Higher LogWorth values indicate stronger statistical evidence against the null hypothesis (i.e., smaller *p*-values), not measures of effect size or the magnitude of biological effects.

**Table 2:** Estimates of the species constants of the seed viability equation (Equation (2)) for the *Lallemantia* species used in this study.

Plant Species	K_E_ (±SE)	C_W_ (±SE)	C_H_ (±SE)	C_Q_ (±SE)
*L. iberica*	2.61 (±0.067)	0.0834 (±0.035)	0.032 (±0.004)	0.0008 (±0.0007)
*L. royleana*	3.91 (±0.290)	1.40 (±0.154)	0.0022 (±0.019)	0.0004 (±0.0003)
*p*-value	<0.001	<0.001	0.12	0.60
Difference (95% CI)	−1.30 (−1.91, −0.69)	−1.3166 (−1.626, −1.007)	0.0298 (−0.0075, 0.0671)	0.0004 (−0.0011, 0.0019)

K_E_: species-specific constant for inherent seed longevity; C_W_: storability response to desiccation; C_H_ and C_Q_: longevity responses to temperature; SE: standard error of the fitted parameter estimates. Difference was calculated as *L. iberica* − *L. royleana*. The standard error of the difference was calculated from the estimated standard errors of the two parameter estimates, assuming independence, and 95% confidence intervals were calculated accordingly. *p*-values indicate the statistical significance of differences between the species-specific parameter estimates.

**Table 3:** Seedling length and seedling dry weight of *L. iberica* and *L. royleana* under different storage treatments.

Traits	Treatments		*L. iberica*			*L. royleana*		
			Storage Temperature			Storage Temperature		
	Storage Duration (Day)	SMC (%)	15 °C	35 °C	45 °C	15 °C	35 °C	45 °C
Seedling length (mm)	0	5	12.4 ± 0.068 c	12.4 ± 0.068 c	12.4 ± 0.068 c	13.42 ± 0.015 a	13.42 ± 0.015 a	13.42 ± 0.015 a
		9	11.6 ± 0.073 d	11.6 ± 0.073 d	11.6 ± 0.073 d	12.85 ± 0.012 b	12.85 ± 0.012 b	12.85 ± 0.012 b
		13	10.94 ± 0.024 f	10.94 ± 0.024 f	10.94 ± 0.024 f	11.59 ± 0.067 d	11.59 ± 0.067 d	11.59 ± 0.067 d
		17	9.49 ± 0.037 i	9.49 ± 0.037 i	9.49 ± 0.037 i	10.94 ± 0.024 f	10.94 ± 0.024 f	10.94 ± 0.024 f
	30	5	12.4 ± 0.068 c	11.6 ± 0.068 d	10.94 ± 0.073 f	13.42 ± 0.015 a	13.28 ± 0.015 a	12.84 ± 0.012 b
		9	11.6 ± 0.068 d	10.94 ± 0.073 f	9.54 ± 0.024 i	12.64 ± 0.068 bc	12.41 ± 0.073 c	11.56 ± 0.009 d
		13	10.94 ± 0.073 f	9.54 ± 0.024 i	9.44 ± 0.037 i	11.29 ± 0.037 e	10.48 ± 0.009 g	10.11 ± 0.04 h
		17	9.54 ± 0.024 i	9.44 ± 0.037 i	8.62 ± 0.091 j	10.48 ± 0.035 g	10.11 ± 0.04 h	9.44 ± 0.037 i
	60	5	11.6 ± 0.068 d	10.94 ± 0.073 f	9.54 ± 0.024 i	12.84 ± 0.015 b	11.56 ± 0.012 d	11.29 ± 0.073 e
		9	10.94 ± 0.073 f	9.54 ± 0.024 i	9.44 ± 0.037 i	11.55 ± 0.012 d	11.28 ± 0.003 e	10.48 ± 0.034 g
		13	9.54 ± 0.024 i	9.44 ± 0.037 i	8.62 ± 0.091 j	11.28 ± 0.003 e	10.11 ± 0.034 h	9.44 ± 0.006 i
		17	9.44 ± 0.037 i	8.62 ± 0.091 j	7.51 ± 0.07 l	10.11 ± 0.034 h	9.44 ± 0.006 i	8.16 ± 0.037 k
	90	5	10.94 ± 0.073 f	9.54 ± 0.024 i	9.49 ± 0.037 i	11.56 ± 0.012 d	11.29 ± 0.073 e	10.94 ± 0.024 f
		9	9.54 ± 0.024 i	8.61 ± 0.037 j	8.62 ± 0.091 j	11.29 ± 0.073 e	10.94 ± 0.024 f	9.49 ± 0.037 i
		13	8.61 ± 0.037 j	8.62 ± 0.091 j	7.5 ± 0.07 l	10.94 ± 0.024 f	9.49 ± 0.037 i	8.16 ± 0.037 k
		17	8.62 ± 0.091 j	7.5 ± 0.07 l	3.83 ± 0.637 m	9.49 ± 0.037 i	8.16 ± 0.037 k	7.5 ± 0.07 l
Seedling weight (mg)	0	5	0.08 ± 0.001 b	0.08 ± 0.001 b	0.08 ± 0.01 b	0.08 ± 0.001 a	0.08 ± 0.001 a	0.08 ± 0.001 a
		9	0.07 ± 0.001 d	0.07 ± 0.001 d	0.07 ± 0.001 d	0.07 ± 0.001 c	0.07 ± 0.001 c	0.07 ± 0.001 c
		13	0.06 ± 0.001 h	0.06 ± 0.001 h	0.06 ± 0.001 h	0.07 ± 0.001 de	0.07 ± 0.001 de	0.07 ± 0.001 de
		17	0.05 ± 0.001 i	0.05 ± 0.001 i	0.05 ± 0.001 i	0.06 ± 0 e.g	0.06 ± 0 e.g	0.06 ± 0 e.g
	30	5	0.08 ± 0.001 b b	0.07 ± 0.001 d	0.06 ± 0.001 e.g	0.08 ± 0.001 ab	0.08 ± 0.001 b	0.07 ± 0.001 d
		9	0.07 ± 0.001 d.f	0.06 ± 0.001 g	0.05 ± 0.001 i	0.07 ± 0.001 c	0.07 ± 0.001 d	0.06 ± 0.001 g
		13	0.06 ± 0.001 g	0.05 ± 0.001 i	0.05 ± 0.0 l	0.06 ± 0.001 fg	0.06 ± 0.001 h	0.05 ± 0.001 ij
		17	0.05 ± 0.001 i.k	0.05 ± 0.01 l	0.04 ± 0.001 m	0.06 ± 0.001 h	0.05 ± 0.001 i	0.05 ± 0 l
	60	5	0.07 ± 0.001 d	0.06 ± 0.001 e.g	0.05 ± 0.001 ij	0.08 ± 0 b	0.07 ± 0.001 d	0.06 ± 0.001 e.g
		9	0.06 ± 0.001 e.g	0.05 ± 0.001 i	0.05 ± 0.001 i.l	0.07 ± 0.001 d	0.06 ± 0.001 e.g	0.05 ± 0.001 i
		13	0.05 ± 0.001 i	0.05 ± 0.001 i.l	0.03 ± 0.002 m	0.06 ± 0.001 e.g	0.05 ± 0.001 i	0.05 ± 0.001 i.l
		17	0.05 ± 0.001 i.l	0.03 ± 0.002 m	0.03 ± 0.001 n	0.05 ± 0.001 i	0.05 ± 0.001 i.l	0.03 ± 0.002 m
	90	5	0.06 ± 0.001 e.g	0.05 ± 0.001 i.k	0.05 ± 0.002 i.l	0.07 ± 0.001 d	0.06 ± 0.001 e.g	0.05 ± 0.001 ij
		9	0.05 ± 0 i.k	0.05 ± 0.001 i.l	0.03 ± 0.002 m	0.06 ± 0.001 e.g	0.05 ± 0.001 ij	0.05 ± 0.001 i.l
		13	0.05 ± 0.001 i.l	0.03 ± 0.002 m	0.03 ± 0.001 n	0.05 ± 0.001 ij	0.05 ± 0.001 i.l	0.03 ± 0.002 m
		17	0.03 ± 0.002 m	0.03 ± 0.001 n	0.02 ± 0.001 o	0.05 ± 0.001 i.l	0.03 ± 0.002 m	0.03 ± 0.001 n

Means followed by the same letter within each species and trait are not significantly different according to Tukey’s multiple range test (*p* < 0.05). Seedling length and seedling dry weight were determined at the end of the germination test (14 days), conducted at 10 °C under a 16/8 h fluorescent-white light/dark cycle and 85% relative humidity. SMC: seed moisture content. For clarity, statistical grouping letters with omitted intermediate letters are presented with a dot (e.g., “a.d” represents “abcd”); the dot has no statistical meaning.

**Table 4:** Electrical conductivity, leachate pH, and O_2_•^−^ of *L. iberaca* and *L. royleana* seeds under different storage treatments.

Traits	Treatments		*L. iberica*			*L. royleana*		
			Storage Temperature			Storage Temperature		
	Storage Duration (Day)	SMC (%)	15 °C	35 °C	45 °C	15 °C	35 °C	45 °C
EC (µS·cm^−1^·g^−1^)	0	5	15.61 ± 0.009 i	15.61 ± 0.009 i	15.61 ± 0.009 i	12.35 ± 1.452 k	12.35 ± 1.452 k	12.35 ± 1.452 k
		9	16 ± 0.007 hi	16 ± 0.007 hi	16 ± 0.007 hi	14.2 ± 0.007 j	14.2 ± 0.007 j	14.2 ± 0.007 j
		13	16.15 ± 0.012 hi	16.15 ± 0.012 hi	16.15 ± 0.012 hi	14.34 ± 0.012 j	14.34 ± 0.012 j	14.34 ± 0.012 j
		17	16.9 ± 0.053 gh	16.9 ± 0.053 gh	16.9 ± 0.053 gh	15.6 ± 0.007 i	15.6 ± 0.007 i	15.6 ± 0.007 i
	30	5	15.61 ± 0.009 i	15.61 ± 0.009 i	15.61 ± 0.009 i	12.35 ± 1.452 k	15.61 ± 0.01 i	16 ± 0.003 hi
		9	16.16 ± 0.01 hi	15.61 ± 0.009 i	15.61 ± 0.009 i	15.59 ± 0.003 i	16 ± 0.009 hi	16.89 ± 0.057 gh
		13	16.82 ± 0.052 gh	17.34 ± 0.006 fg	18.21 ± 0.012 ef	16 ± 0.007 hi	16.89 ± 0.053 gh	17.32 ± 0.003 fg
		17	17.34 ± 0.006 fg	18.21 ± 0.015 ef	18.43 ± 0.015 c.e	16.89 ± 0.05 gh	17.32 ± 0.006 fg	17.66 ± 0.029 e.g
	60	5	16.16 ± 0.017 hi	16.83 ± 0.047 gh	17.35 ± 0.006 fg	14.35 ± 0.013 j	15.74 ± 0.047 i	16.13 ± 0.017 hi
		9	16.83 ± 0.047 gh	17.35 ± 0.006 fg	18.22 ± 0.012 ef	15.74 ± 0.047 i	16.13 ± 0.017 hi	17.32 ± 0.006 fg
		13	17.35 ± 0.006 fg	18.22 ± 0.012 ef	19.41 ± 0.027 bc	16.13 ± 0.017 hi	17.32 ± 0.006 fg	18.19 ± 0.012 ef
		17	18.22 ± 0.012 ef	19.41 ± 0.027 bc	19.8 ± 0.061 b	17.32 ± 0.006 fg	18.19 ± 0.012 ef	19.32 ± 0.009 b.d
	90	5	16.82 ± 0.047 gh	17.34 ± 0.009 fg	18.21 ± 0.012 ef	15.74 ± 0.047 i	16.13 ± 0.017 hi	17.32 ± 0.006 fg
		9	17.33 ± 0.012 fg	18.21 ± 0.015 ef	19.39 ± 0.021 b.d	16.13 ± 0.017 hi	17.32 ± 0.006 fg	18.19 ± 0.012 ef
		13	18.21 ± 0.003 ef	19.39 ± 0.03 b.d	19.78 ± 0.059 b	17.32 ± 0.006 fg	18.19 ± 0.012 ef	18.32 ± 0.012 ef
		17	19.4 ± 0.026 b.d	19.79 ± 0.062 b	23.77 ± 0.876 a	18.19 ± 0.012 ef	18.32 ± 0.012 ef	19.38 ± 0.027 b.d
pH	0	5	5.81 ± 0.198 c	5.81 ± 0.198 c	5.81 ± 0.198 c	6.78 ± 0.009 a	6.78 ± 0.009 a	6.78 ± 0.009 a
		9	5.25 ± 0.065 d	5.25 ± 0.065 d	5.25 ± 0.065 d	6.41 ± 0.022 b	6.41 ± 0.022 b	6.41 ± 0.022 b
		13	5.1 ± 0.009 de	5.1 ± 0.009 de	5.1 ± 0.009 de	5.82 ± 0.198 c	5.82 ± 0.198 c	5.82 ± 0.198 c
		17	4.89 ± 0.049 e	4.89 ± 0.049 e	4.89 ± 0.049 e	5.11 ± 0.009 de	5.11 ± 0.009 de	5.11 ± 0.009 de
	30	5	5.81 ± 0.198 c	5.09 ± 0.009 de	4.88 ± 0.049 e	6.78 ± 0.009 a	5.81 ± 0.198 c	5.09 ± 0.009 de
		9	5.09 ± 0.009 de	4.88 ± 0.049 e	4.6 ± 0.026 f	5.81 ± 0.198 c	5.09 ± 0.009 de	4.88 ± 0.049 e
		13	4.88 ± 0.049 e	4.6 ± 0.026 f	4.33 ± 0.022 g	5.09 ± 0.009 de	4.88 ± 0.049 e	4.6 ± 0.026 f
		17	4.63 ± 0.009 f	4.33 ± 0.022 g	3.82 ± 0.012 h	4.88 ± 0.049 e	4.6 ± 0.026 f	4.33 ± 0.022 g
	60	5	5.11 ± 0.009 de	4.9 ± 0.049 e	4.62 ± 0.026 f	5.82 ± 0.198 c	5.1 ± 0.009 de	4.89 ± 0.049 e
		9	4.9 ± 0.049 e	4.62 ± 0.026 f	4.35 ± 0.022 g	5.1 ± 0.009 de	4.89 ± 0.049 e	4.61 ± 0.026 f
		13	4.62 ± 0.026 f	4.35 ± 0.022 g	3.85 ± 0.018 h	4.89 ± 0.049 e	4.61 ± 0.026 f	4.34 ± 0.022 g
		17	4.35 ± 0.022 g	3.85 ± 0.018 h	3.58 ± 0.009 i	4.61 ± 0.026 f	4.34 ± 0.022 g	3.84 ± 0.018 h
	90	5	4.88 ± 0.049 e	4.6 ± 0.026 f	4.33 ± 0.022 g	5.1 ± 0.012 de	4.88 ± 0.047 e	4.61 ± 0.026 f
		9	4.6 ± 0.026 f	4.33 ± 0.022 g	3.83 ± 0.018 h	4.88 ± 0.047 e	4.61 ± 0.026 f	4.34 ± 0.022 g
		13	4.33 ± 0.022 g	3.83 ± 0.018 h	3.56 ± 0.009 i	4.61 ± 0.026 f	4.31 ± 0.006 g	3.84 ± 0.018 h
		17	3.83 ± 0.018 h	3.56 ± 0.009 i	2.46 ± 0.264 j	4.31 ± 0.006 g	3.84 ± 0.018 h	3.55 ± 0.009 i
O_2_•^−^ (µmol g^−1^)	0	5	4.56 ± 0.009 i	4.56 ± 0.009 i	4.56 ± 0.009 i	3.75 ± 0.061 k	3.75 ± 0.015 k	3.75 ± 0.009 k
		9	4.78 ± 0.067 h	4.78 ± 0.067 h	4.78 ± 0.067 h	3.99 ± 0.015 j	3.99 ± 0.009 j	3.99 ± 0.067 j
		13	5.38 ± 0.04 g	5.38 ± 0.04 g	5.38 ± 0.04 g	4.53 ± 0.009 i	4.53 ± 0.067 i	4.53 ± 0.04 i
		17	5.98 ± 0.019 f	5.98 ± 0.019 f	5.98 ± 0.019 f	4.75 ± 0.067 h	4.75 ± 0.04 h	4.75 ± 0.042 h
	30	5	4.56 ± 0.009 i	5.37 ± 0.04 g	5.97 ± 0.019 f	3.75 ± 0.061 k	4.57 ± 0.009 i	4.79 ± 0.067 h
		9	5.37 ± 0.04 g	5.97 ± 0.019 f	6.47 ± 0.042 e	4.57 ± 0.009 i	4.79 ± 0.067 h	5.39 ± 0.04 g
		13	5.97 ± 0.019 f	6.47 ± 0.042 e	7.17 ± 0.03 d	4.79 ± 0.067 h	5.39 ± 0.04 g	5.99 ± 0.019 f
		17	6.47 ± 0.042 e	7.17 ± 0.03 d	7.44 ± 0.015 c	5.39 ± 0.04 g	5.99 ± 0.019 f	6.49 ± 0.042 e
	60	5	5.36 ± 0.04 g	5.96 ± 0.019 f	5.98 ± 0.019 f	4.58 ± 0.009 i	4.8 ± 0.067 h	5.38 ± 0.04 g
		9	5.96 ± 0.019 f	5.98 ± 0.019 f	6.46 ± 0.042 e	4.8 ± 0.067 h	5.38 ± 0.04 g	6.48 ± 0.042 e
		13	5.98 ± 0.019 f	6.46 ± 0.042 e	7.16 ± 0.03 d	5.38 ± 0.04 g	6.48 ± 0.042 e	7.18 ± 0.03 d
		17	7.16 ± 0.03 d	7.43 ± 0.015 c	7.97 ± 0.073 b	6.48 ± 0.042 e	7.18 ± 0.03 d	7.44 ± 0.015 c
	90	5	5.94 ± 0.019 f	5.96 ± 0.019 f	6.44 ± 0.042 e	4.81 ± 0.067 h	5.39 ± 0.04 g	5.99 ± 0.019 f
		9	5.96 ± 0.019 f	6.44 ± 0.042 e	7.14 ± 0.03 d	5.39 ± 0.04 g	5.99 ± 0.019 f	6.47 ± 0.042 e
		13	6.44 ± 0.042 e	7.14 ± 0.03 d	7.95 ± 0.073 b	5.99 ± 0.019 f	6.47 ± 0.042 e	7.15 ± 0.03 d
		17	7.14 ± 0.03 d	7.95 ± 0.073 b	9.42 ± 0.069 a	6.47 ± 0.042 e	7.15 ± 0.03 d	7.44 ± 0.015 c

Means followed by the same letter within each species and trait are not significantly different according to Tukey’s multiple range test (*p* < 0.05). EC, pH, and O_2_•^−^ were determined in seeds immediately after the respective storage treatments. For clarity, statistical grouping letters with omitted intermediate letters are presented with a dot (e.g., “a.d” represents “abcd”); the dot has no statistical meaning. SMC: seed moisture content; EC: electrical conductivity; O_2_•^−^: superoxide anion.

**Table 5:** Catalase and ascorbate peroxidase enzyme activities of *L. iberica* and *L. royleana* seeds under different storage treatments.

Traits	Treatments		*L. iberica*			*L. royleana*		
			Storage Temperature			Storage Temperature		
	Storage Duration (Day)	SMC (%)	15 °C	35 °C	45 °C	15 °C	35 °C	45 °C
CAT (Unit mg^−1^ protein min^−1^)	0	5	5.36 ± 0.033 cd	5.36 ± 0.009 cd	5.36 ± 0.022 cd	6.8 ± 0.423 a	6.8 ± 0.423 a	6.8 ± 0.033 a
		9	5.11 ± 0.009 de	5.11 ± 0.022 de	5.11 ± 0.03 de	5.58 ± 0.006 bc	5.58 ± 0.006 bc	5.58 ± 0.009 bc
		13	4.83 ± 0.022 ef	4.83 ± 0.03 ef	4.83 ± 0.012 ef	5.39 ± 0.033 cd	5.39 ± 0.033 cd	4.85 ± 0.022 ef
		17	4.71 ± 0.03 f	4.71 ± 0.012 f	4.71 ± 0.015 f	5.13 ± 0.009 de	5.13 ± 0.009 de	5.13 ± 0.03 de
	30	5	5.36 ± 0.033 cd	4.85 ± 0.022 ef	4.73 ± 0.03 f	6.79 ± 0.432 a	5.69 ± 0.348 b	5.12 ± 0.009 de
		9	4.85 ± 0.022 ef	4.73 ± 0.03 f	4.32 ± 0.012 g	5.38 ± 0.033 cd	5.12 ± 0.009 de	4.7 ± 0.03 f
		13	4.73 ± 0.03 f	4.32 ± 0.012 g	3.93 ± 0.015 h	5.12 ± 0.009 de	4.7 ± 0.03 f	4.29 ± 0.012 g
		17	4.32 ± 0.012 g	3.93 ± 0.015 h	3.43 ± 0.015 i	4.7 ± 0.03 f	4.29 ± 0.012 g	3.9 ± 0.015 h
	60	5	4.84 ± 0.022 ef	4.72 ± 0.03 f	4.31 ± 0.012 g	5.37 ± 0.033 cd	5.11 ± 0.009 de	4.69 ± 0.03 f
		9	4.72 ± 0.03 f	4.31 ± 0.012 g	3.92 ± 0.015 h	5.11 ± 0.009 de	4.69 ± 0.03 f	4.28 ± 0.012 g
		13	4.31 ± 0.012 g	3.92 ± 0.015 h	3.42 ± 0.015 i	4.69 ± 0.03 f	4.28 ± 0.012 g	3.92 ± 0.015 h
		17	3.92 ± 0.015 h	3.42 ± 0.015 i	2.88 ± 0.026 j	4.28 ± 0.012 g	3.92 ± 0.015 h	3.42 ± 0.015 i
	90	5	4.74 ± 0.03 f	4.33 ± 0.012 g	3.94 ± 0.015 h	5.1 ± 0.009 de	4.68 ± 0.03 f	4.27 ± 0.012 g
		9	4.33 ± 0.012 g	3.94 ± 0.015 h	2.89 ± 0.026 j	4.68 ± 0.03 f	4.27 ± 0.012 g	3.91 ± 0.015 h
		13	3.94 ± 0.015 h	2.89 ± 0.026 j	2.27 ± 0.012 k	4.27 ± 0.012 g	3.91 ± 0.015 h	3.41 ± 0.015 i
		17	2.89 ± 0.026 j	2.27 ± 0.012 k	1.66 ± 0.069 l	3.91 ± 0.015 h	3.41 ± 0.015 i	2.87 ± 0.026 j
APX (Unit mg^−1^ protein min^−1^)	0	5	3.87 ± 0.035 d	3.87 ± 0.035 d	3.87 ± 0.035 d	4.83 ± 0.018 a	4.83 ± 0.018 a	4.83 ± 0.018 a
		9	3.53 ± 0.015 e	3.53 ± 0.015 e	3.53 ± 0.015 e	4.62 ± 0.015 b	4.62 ± 0.015 b	4.62 ± 0.015 b
		13	3.11 ± 0.009 f	3.11 ± 0.009 f	3.11 ± 0.009 f	4.12 ± 0.012 c	4.12 ± 0.012 c	4.12 ± 0.012 c
		17	2.85 ± 0.006 g	2.85 ± 0.006 g	2.85 ± 0.006 g	3.87 ± 0.035 d	3.87 ± 0.035 d	3.87 ± 0.035 d
	30	5	3.87 ± 0.035 d	3.12 ± 0.009 f	2.86 ± 0.006 g	4.83 ± 0.018 a	4.1 ± 0.012 c	3.85 ± 0.035 d
		9	3.12 ± 0.009 f	2.86 ± 0.006 g	2.7 ± 0.018 h	4.1 ± 0.012 c	3.85 ± 0.035 d	3.51 ± 0.015 e
		13	2.86 ± 0.006 g	2.7 ± 0.018 h	2.52 ± 0.023 i	3.85 ± 0.035 d	3.51 ± 0.015 e	3.09 ± 0.009 f
		17	2.7 ± 0.018 h	2.52 ± 0.023 i	2.16 ± 0.018 j	3.51 ± 0.015 e	3.09 ± 0.009 f	2.83 ± 0.006 g
	60	5	3.13 ± 0.009 f	2.87 ± 0.006 g	2.71 ± 0.018 h	4.09 ± 0.012 c	3.84 ± 0.035 d	3.11 ± 0.009 f
		9	2.87 ± 0.006 g	2.71 ± 0.018 h	2.53 ± 0.023 i	3.84 ± 0.035 d	3.11 ± 0.009 f	2.85 ± 0.006 g
		13	2.71 ± 0.018 h	2.53 ± 0.023 i	2.17 ± 0.018 j	3.11 ± 0.009 f	2.85 ± 0.006 g	2.69 ± 0.018 h
		17	2.53 ± 0.023 i	2.17 ± 0.018 j	1.86 ± 0.017 k	2.85 ± 0.006 g	2.69 ± 0.018 h	2.51 ± 0.023 i
	90	5	2.88 ± 0.006 g	2.72 ± 0.018 h	2.54 ± 0.023 i	3.83 ± 0.035 d	3.1 ± 0.009 f	2.86 ± 0.006 g
		9	2.72 ± 0.018 h	2.54 ± 0.023 i	2.18 ± 0.018 j	3.1 ± 0.009 f	2.86 ± 0.006 g	2.7 ± 0.018 h
		13	2.54 ± 0.023 i	2.18 ± 0.018 j	1.87 ± 0.017 k	2.86 ± 0.006 g	2.7 ± 0.018 h	2.16 ± 0.018 j
		17	2.18 ± 0.018 j	1.87 ± 0.017 k	1.52 ± 0.021 l	2.7 ± 0.018 h	2.16 ± 0.018 j	1.85 ± 0.017 k

Means followed by the same letter within each species and trait are not significantly different according to Tukey’s multiple range test (*p* < 0.05). CAT and APX were determined in seeds immediately after the respective storage treatments. SMC: seed moisture content; CAT: catalase; APX: ascorbate peroxidase. For clarity, statistical grouping letters with omitted intermediate letters are presented with a dot (e.g., “a.d” represents “abcd”); the dot has no statistical meaning.

**Table 6:** Unsaturated and saturated fatty acid contents in *L. iberica* and *L. royleana* seeds under different storage treatments.

Traits	Treatments		*L. iberica*			*L. royleana*		
			Storage Temperature			Storage Temperature		
	Storage Duration (Day)	SMC (%)	15 °C	35 °C	45 °C	15 °C	35 °C	45 °C
Linoleic acid (%)	0	5	54.57 ± 0.209 c	54.57 ± 0.209 c	54.57 ± 0.209 c	58.4 ± 1.286 a	58.4 ± 1.286 a	58.4 ± 1.286 a
		9	53.37 ± 0.128 c.e	53.37 ± 0.128 c.e	53.37 ± 0.128 c.e	56.37 ± 0.289 b	56.37 ± 0.289 b	56.37 ± 0.289 b
		13	52.29 ± 0.151 ef	52.29 ± 0.151 ef	52.29 ± 0.151 ef	54.83 ± 0.209 bc	54.83 ± 0.209 bc	54.83 ± 0.209 bc
		17	51.23 ± 0.403 fg	51.23 ± 0.403 fg	51.23 ± 0.403 fg	53.63 ± 0.128 c.e	53.63 ± 0.128 c.e	53.63 ± 0.128 c.e
	30	5	54.57 ± 0.209 c	51.27 ± 0.151 fg	50.21 ± 0.403 gh	58.4 ± 1.286 a	54.15 ± 0.396 c	52.39 ± 0.128 d.f
		9	51.27 ± 0.151 fg	50.21 ± 0.403 gh	48.71 ± 0.199 h.j	54.15 ± 0.396 c	52.39 ± 0.128 d.f	51.31 ± 0.151 fg
		13	50.21 ± 0.403 gh	48.71 ± 0.199 h.j	47.53 ± 0.199 j.l	52.39 ± 0.128 d.f	51.31 ± 0.151 fg	50.25 ± 0.403 gh
		17	48.71 ± 0.199 h.j	47.53 ± 0.199 j.l	46.71 ± 0.149 k.m	51.31 ± 0.151 fg	50.25 ± 0.403 gh	48.75 ± 0.199 h.j
	60	5	51.06 ± 0.151 fg	50 ± 0.403 g.i	48.5 ± 0.199 h.j	54.04 ± 0.396 cd	52.28 ± 0.128 ef	51.16 ± 0.151 fg
		9	50 ± 0.403 g.i	48.5 ± 0.199 h.j	47.32 ± 0.199 j.l	52.28 ± 0.128 ef	51.16 ± 0.151 fg	50.1 ± 0.403 g.i
		13	48.5 ± 0.199 h.j	47.32 ± 0.199 j.l	46.5 ± 0.149 lm	51.16 ± 0.151 fg	50.1 ± 0.403 g.i	48.6 ± 0.199 h.j
		17	47.32 ± 0.199 j.l	46.5 ± 0.149 lm	45.36 ± 0.132 m	50.1 ± 0.403 g.i	48.6 ± 0.199 h.j	47.42 ± 0.199 j.l
	90	5	51.16 ± 0.151 fg	50.1 ± 0.403 g.i	48.6 ± 0.199 h.j	52.19 ± 0.128 ef	51.07 ± 0.151 fg	50.01 ± 0.403 g.i
		9	50.1 ± 0.403 g.i	48.6 ± 0.199 h.j	47.42 ± 0.199 j.l	51.07 ± 0.151 fg	50.01 ± 0.403 g.i	48.41 ± 0.199 i.l
		13	48.6 ± 0.199 h.j	47.42 ± 0.199 j.l	46.6 ± 0.149 lm	50.01 ± 0.403 g.i	48.41 ± 0.199 i.k	47.23 ± 0.199 j.l
		17	47.42 ± 0.199 j.l	46.6 ± 0.149 lm	35.9 ± 0.876 n	48.41 ± 0.199 i.k	47.23 ± 0.199 j.l	46.41 ± 0.149 lm
Linolenic acid (%)	0	5	13.26 ± 0.276 c	13.26 ± 0.276 c	13.26 ± 0.276 c	16.16 ± 0.718 a	16.16 ± 0.718 a	16.16 ± 0.718 a
		9	11.67 ± 0.095 de	11.67 ± 0.095 de	11.67 ± 0.095 de	13.26 ± 0.276 c	13.26 ± 0.276 c	13.26 ± 0.276 c
		13	10.59 ± 0.121 fgh	10.59 ± 0.121 fgh	10.59 ± 0.121 fgh	11.91 ± 0.121 d	11.91 ± 0.121 d	11.91 ± 0.121 d
		17	9.84 ± 0.044 i	9.84 ± 0.044 i	9.84 ± 0.044 i	11.16 ± 0.044 e.g	11.16 ± 0.044 e.g	11.16 ± 0.044 e.g
	30	5	13.26 ± 0.276 c	10.56 ± 0.121 f.h	9.81 ± 0.044 i	16.16 ± 0.718 a	14.55 ± 0.143 b	13.23 ± 0.276 c
		9	10.56 ± 0.121 f.h	9.81 ± 0.044 i	9.06 ± 0.055 j	14.55 ± 0.143 b	13.23 ± 0.276 c	11.64 ± 0.095 de
		13	9.81 ± 0.044 i	9.06 ± 0.055 j	8.23 ± 0.046 k	13.23 ± 0.276 c	11.64 ± 0.095 de	10.56 ± 0.121 f.h
		17	9.06 ± 0.055 j	8.23 ± 0.046 k	7.65 ± 0.069 kl	11.64 ± 0.095 de	10.56 ± 0.121 f.h	9.81 ± 0.044 i
	60	5	10.54 ± 0.121 f.h	9.79 ± 0.044 i	9.04 ± 0.055 j	14.53 ± 0.143 b	13.21 ± 0.276 c	11.39 ± 0.034 de
		9	9.79 ± 0.044 i	9.04 ± 0.055 j	8.21 ± 0.046 k	13.21 ± 0.276 c	11.39 ± 0.034 de	10.54 ± 0.121 f.h
		13	9.04 ± 0.055 j	8.21 ± 0.046 k	7.63 ± 0.069 kl	11.39 ± 0.034 de	10.54 ± 0.121 f.h	9.79 ± 0.044 i
		17	8.21 ± 0.046 k	7.63 ± 0.069 kl	6.65 ± 0.112 m	10.54 ± 0.121 f.h	9.79 ± 0.044 i	8.21 ± 0.046 k
	90	5	9.69 ± 0.044 i	8.94 ± 0.055 j	8.11 ± 0.046 kl	13.18 ± 0.276 c	11.36 ± 0.034 de	10.51 ± 0.121 f h
		9	8.94 ± 0.055 j	8.11 ± 0.046 kl	7.53 ± 0.069 l	11.36 ± 0.034 de	10.51 ± 0.121 f.h	9.01 ± 0.055 j
		13	8.11 ± 0.046 kl	7.53 ± 0.069 l	6.55 ± 0.112 m	10.51 ± 0.121 f h	9.01 ± 0.055 j	7.6 ± 0.069 kl
		17	7.53 ± 0.069 l	6.55 ± 0.112 m	5.01 ± 0.127 n	9.01 ± 0.055 j	7.6 ± 0.069 kl	6.62 ± 0.112 m
Oleic acid (%)	0	5	14.74 ± 0.03 c	14.74 ± 0.03 c	14.74 ± 0.03 c	16.76 ± 0.096 a	16.76 ± 0.096 a	16.76 ± 0.096 a
		9	13.34 ± 0.012 d	13.34 ± 0.012 d	13.34 ± 0.012 d	14.97 ± 0.03 b	14.97 ± 0.03 b	14.97 ± 0.03 b
		13	12.47 ± 0.127 e	12.47 ± 0.127 e	12.47 ± 0.127 e	14.77 ± 0.03 bc	14.77 ± 0.03 bc	14.77 ± 0.03 bc
		17	11.89 ± 0.061 f	11.89 ± 0.061 f	11.89 ± 0.061 f	13.37 ± 0.012 d	13.37 ± 0.012 d	13.37 ± 0.012 d
	30	5	14.74 ± 0.03 c	12.46 ± 0.127 e	11.88 ± 0.061 f	16.76 ± 0.096 a	14.79 ± 0.079 bc	13.33 ± 0.012 d
		9	12.46 ± 0.127 e	11.88 ± 0.061 f	10.86 ± 0.044 h	14.79 ± 0.079 bc	13.33 ± 0.012 d	12.43 ± 0.127 e
		13	11.88 ± 0.061 f	10.86 ± 0.044 h	10.11 ± 0.017 i	13.33 ± 0.012 d	12.43 ± 0.127 e	11.85 ± 0.061 f
		17	10.86 ± 0.044 h	10.11 ± 0.017 i	9.79 ± 0.023 j	12.43 ± 0.127 e	11.85 ± 0.061 f	10.83 ± 0.044 h
	60	5	12.44 ± 0.127 e	11.86 ± 0.061 f	10.84 ± 0.044 h	14.75 ± 0.079 c	13.29 ± 0.012 d	12.39 ± 0.127 e
		9	11.86 ± 0.061 f	10.84 ± 0.044 h	9.78 ± 0.023 j	13.29 ± 0.012 d	12.39 ± 0.127 e	11.33 ± 0.038 g
		13	10.84 ± 0.044 h	9.78 ± 0.023 j	8.56 ± 0.096 k	12.39 ± 0.127 e	11.33 ± 0.038 g	10.83 ± 0.044 h
		17	9.78 ± 0.023 j	8.56 ± 0.096 k	7.9 ± 0.035 l	11.33 ± 0.038 g	10.83 ± 0.044 h	10.12 ± 0.017 i
	90	5	11.85 ± 0.061 f	10.83 ± 0.044 h	9.77 ± 0.023 j	13.24 ± 0.012 d	12.34 ± 0.127 e	11.28 ± 0.038 g
		9	10.83 ± 0.044 h	9.77 ± 0.023 j	8.55 ± 0.096 k	12.34 ± 0.127 e	11.28 ± 0.038 g	10.82 ± 0.044 h
		13	9.77 ± 0.023 j	8.55 ± 0.096 k	7.89 ± 0.035 l	11.28 ± 0.038 g	10.82 ± 0.044 h	10.07 ± 0.017 i
		17	8.55 ± 0.096 k	7.89 ± 0.035 l	6.69 ± 0.2 m	10.82 ± 0.044 h	10.07 ± 0.017 i	9.75 ± 0.023 j
Palmitic acid (%)	0	5	5.44 ± 0.009 i	5.44 ± 0.009 i	5.44 ± 0.009 i	3.57 ± 0.04 k	3.57 ± 0.04 k	3.57 ± 0.04 k
		9	6.84 ± 0.037 h	6.84 ± 0.037 h	6.84 ± 0.037 h	4.32 ± 0.015 j	4.32 ± 0.015 j	4.32 ± 0.015 j
		13	7.19 ± 0.017 gh	7.19 ± 0.017 gh	7.19 ± 0.017 gh	5.42 ± 0.009 i	5.42 ± 0.009 i	5.42 ± 0.009 i
		17	7.34 ± 0.006 g	7.34 ± 0.006 g	7.34 ± 0.006 g	6.82 ± 0.037 h	6.82 ± 0.037 h	6.82 ± 0.037 h
	30	5	5.44 ± 0.009 i	7.2 ± 0.017 gh	7.35 ± 0.006 g	3.58 ± 0.04 k	5.43 ± 0.009 i	6.85 ± 0.037 h
		9	7.2 ± 0.017 gh	7.35 ± 0.006 g	7.88 ± 0.072 de	5.43 ± 0.009 i	6.85 ± 0.037 h	7.2 ± 0.017 gh
		13	7.35 ± 0.006 g	7.88 ± 0.072 de	8.11 ± 0.114 d	6.85 ± 0.037 h	7.2 ± 0.017 gh	7.88 ± 0.072 de
		17	7.88 ± 0.072 d.f	8.11 ± 0.114 d	8.8 ± 0.084 c	7.2 ± 0.017 gh	7.88 ± 0.072 de	8.11 ± 0.114 d
	60	5	7.21 ± 0.017 gh	7.36 ± 0.006 g	7.89 ± 0.072 de	5.42 ± 0.009 i	6.84 ± 0.037 h	7.19 ± 0.017 gh
		9	7.36 ± 0.006 g	7.89 ± 0.072 de	8.03 ± 0.154 d	6.84 ± 0.037 h	7.19 ± 0.017 gh	7.34 ± 0.006 g
		13	7.89 ± 0.072 de	8.03 ± 0.154 d	8.81 ± 0.084 c	7.19 ± 0.017 gh	7.34 ± 0.006 g	7.87 ± 0.072 d.f
		17	8.03 ± 0.154 d	8.81 ± 0.084 c	9.68 ± 0.058 b	7.34 ± 0.006 g	7.87 ± 0.072 d.f	8.1 ± 0.114 d
	90	5	7.35 ± 0.006 g	7.88 ± 0.072 d.f	8.02 ± 0.156 d	6.82 ± 0.037 h	7.17 ± 0.017 gh	7.49 ± 0.148 e g
		9	7.88 ± 0.072 d.f	8.02 ± 0.156 d	8.68 ± 0.188 c	7.17 ± 0.017 gh	7.49 ± 0.148 e.g	8.01 ± 0.154 d
		13	8.02 ± 0.156 d	8.68 ± 0.188 c	9.67 ± 0.058 b	7.49 ± 0.148 e.g	8.01 ± 0.154 d	8.79 ± 0.084 c
		17	8.68 ± 0.188 c	9.67 ± 0.058 b	11.89 ± 0.898 a	8.01 ± 0.154 d	8.79 ± 0.084 c	9.66 ± 0.058 b
Stearic acid (%)	0	5	0.76 ± 0.015 i	0.76 ± 0.015 i	0.76 ± 0.015 i	0.57 ± 0.015 j	0.57 ± 0.015 j	0.57 ± 0.015 j
		9	0.86 ± 0.015 h	0.86 ± 0.015 h	0.86 ± 0.015 h	0.75 ± 0.015 i	0.75 ± 0.015 i	0.75 ± 0.015 i
		13	0.95 ± 0.015 g	0.95 ± 0.015 g	0.95 ± 0.015 g	0.85 ± 0.015 h	0.85 ± 0.015 h	0.85 ± 0.015 h
		17	1.1 ± 0.009 f	1.1 ± 0.009 f	1.1 ± 0.009 f	0.94 ± 0.015 g	0.94 ± 0.015 g	0.94 ± 0.015 g
	30	5	0.76 ± 0.015 i	0.97 ± 0.015 g	1.12 ± 0.009 f	0.57 ± 0.015 j	0.86 ± 0.015 h	0.96 ± 0.015 g
		9	0.97 ± 0.015 g	1.12 ± 0.009 f	1.25 ± 0.02 e	0.86 ± 0.015 h	0.96 ± 0.015 g	1.11 ± 0.009 f
		13	1.12 ± 0.009 f	1.25 ± 0.02 e	1.36 ± 0.015 d	0.96 ± 0.015 g	1.11 ± 0.009 f	1.24 ± 0.02 e
		17	1.25 ± 0.02 e	1.36 ± 0.015 d	1.48 ± 0.015 c	1.11 ± 0.009 f	1.24 ± 0.02 e	1.35 ± 0.015 d
	60	5	0.98 ± 0.015 g	1.13 ± 0.009 f	1.26 ± 0.02 e	0.88 ± 0.015 h	0.98 ± 0.015 g	0.99 ± 0.015 g
		9	1.13 ± 0.009 f	1.26 ± 0.02 e	1.37 ± 0.015 d	0.98 ± 0.015 g	0.99 ± 0.015 g	1.14 ± 0.009 f
		13	1.26 ± 0.02 e	1.37 ± 0.015 d	1.49 ± 0.015 c	0.99 ± 0.015 g	1.14 ± 0.009 f	1.27 ± 0.02 e
		17	1.37 ± 0.015 d	1.49 ± 0.015 c	1.62 ± 0.021 b	1.14 ± 0.009 f	1.27 ± 0.02 e	1.38 ± 0.015 d
	90	5	1.12 ± 0.009 f	1.25 ± 0.02 e	1.36 ± 0.015 d	0.97 ± 0.015 g	0.98 ± 0.015 g	1.12 ± 0.009 f
		9	1.25 ± 0.02 e	1.36 ± 0.015 d	1.48 ± 0.015 c	0.98 ± 0.015 g	1.12 ± 0.009 f	1.25 ± 0.02 e
		13	1.36 ± 0.015 d	1.48 ± 0.015 c	1.61 ± 0.021 b	1.12 ± 0.009 f	1.25 ± 0.02 e	1.36 ± 0.015 d
		17	1.48 ± 0.015 c	1.61 ± 0.021 b	1.95 ± 0.027 a	1.25 ± 0.02 e	1.36 ± 0.015 d	1.48 ± 0.015 c

Means followed by the same letter within each species and trait are not significantly different according to Tukey’s multiple range test (p < 0.05). Fatty acids were determined in seeds immediately after the respective storage treatments. SMC: seed moisture content. For clarity, statistical grouping letters with omitted intermediate letters are presented with a dot (e.g., “a.d” represents “abcd”); the dot has no statistical meaning.

**Table 7:** Characteristics of the seed lots of *L. iberica* and *L. royleana* used in this study.

Seed Quality Parameter	*L. iberica*	*L. royleana*
1000-seed weight (g)	3.9	1.8
Initial germination (%)	99.4	98.7
Moisture content (%)	5.76	7
Seed vigor index	1197.23	1309.85
Seed viability (%)	100	99
Seed Health	Healthy	Healthy
Prior storage history	−28 °C for 1 month	−28 °C for 1 month

Seed health: healthy seeds free from pests and visible disease symptoms. Seed lots were stored at −28 °C for one month before experimental use.

## Data Availability

The raw data supporting the findings of this study are available from the corresponding authors upon reasonable request.

## Supplementary Materials

The following supporting information can be downloaded at: https://www.mdpi.com/article/10.3390/plants15213435/s1, Table S1: Analysis of variance for the effects of storage duration, seed moisture content, storage temperature, and plant species, and their interactions on germination parameters and seedling growth traits; Table S2: Analysis of variance for the effects of storage duration, seed moisture content, storage temperature, and plant species, and their interactions on oxidative products and antioxidant and non-antioxidant enzyme activity; Table S3: Analysis of variance for the effects of storage duration, seed moisture content, storage temperature, and plant species, and their interactions on seed storage compounds; Table S4: LogWorth values indicating the statistical significance of storage factors and their interactions for seed quality of *L. iberica* and *L. royleana*; Table S5: LogWorth values indicating the statistical significance of storage factors and their interactions for respiration rate and oxidative products of *L. iberica* and *L. royleana*; Table S6: LogWorth values indicating the statistical significance of storage factors and their interactions for antioxidant enzyme activities of *L. iberica* and *L. royleana;* Table S7: LogWorth values indicating the statistical significance of storage factors and their interactions for oil, fatty acids, and total soluble sugar of *L. iberica* and *L. royleana*; Table S8: LogWorth values indicating the statistical significance of storage factors and their interactions for storage compounds of *L. iberica* and *L. royleana*.

## Abbreviations

GR: germination rate; MGT: mean germination time; VI: vigor index; SL: seedling length; SW: seedling dry weight; SOD: superoxide dismutase; CAT: catalase; APX: ascorbate peroxidase; To: tocopherol; O_2_•^−^: superoxide anion; H_2_O_2_: hydrogen peroxide; MDA: malondialdehyde; TSS: total soluble sugar; ST: starch; SU: sucrose; Glu: glucose; Fruc: fructose; Res: respiration rate; SP: soluble protein; TFA: total free amino acid.
