# Supplementary material for: Storage Temperature and Moisture Content Differentially Affect Seed Viability, Biochemical Composition, and Oxidative Status in Lallemantia Seeds
Source: Plants (Basel). 2026 Nov 4;15(21):3435. doi: 10.3390/plants15213435 (PMC13765660; doi:10.3390/plants15213435)
Supplement: Supplementary file 1 [file plants-15-03435-s001.zip › plants-4591266-supplementary.pdf]

Table S1. Analysis of variance for the effects of storage duration, seed moisture content, storage temperature, and plant species, and their interactions on germination parameters and seedling growth traits.

Source of variation	DF	GR	MGT	VI	SL	SDW
Storage duration (D)	3	0.005**	100.41**	1128361.248**	102.12**	0.007643**
Moisture content (M)	3	0.002**	29.11**	565235.592**	34.11**	0.003031**
Storage temperature (T)	2	0.005**	140.11**	1083916.832**	101.01**	0.003935**
Plant species (S)	1	0.000174**	1.8**	5241.721**	0.48**	0.000017**
D×M	9	0.000266**	3.54**	62905.338**	4.3**	0.00036**
D×T	6	0.000118**	3**	6931.688**	0.75**	0.000028**
D×S	3	0.000008**	1.07**	337.398**	0.6**	0.000005n.s
M×T	6	0.000068**	1.72**	803.576**	0.71**	0.000022**
M×S	3	0.000002n.s	0.09n.s	337.447*	0.24**	0.000011*
T×S	2	0.000021**	1.89**	1236.217**	0.61**	0.000006**
D×M×T	18	0.000015**	1.28**	2230.66**	0.42**	0.000021**
D×M×S	9	0.000024**	0.27**	413.861**	0.11**	0.000008**
D×T×S	6	0.000021**	1.05**	2623.674**	0.75**	0.000005*
M×T×S	6	0.00002**	1.6**	2395.618**	0.7**	0.000039**
D×M×T×S	18	1E-06	0.06475	105.3	0.0193	0.000002
Error	192	1.47	1.4	1.09	1.32	2.78
CV		0.005**	100.41**	1128361.248**	102.12**	0.007643**

ns, *, and **, not significant and significant at 5 and 1% probability levels, respectively. Each dependent variable was analyzed separately using ANOVA; each column presents the corresponding ANOVA results for the respective trait. Df: degree of freedom; GR: germination rate; MGT: mean germination time; VI: vigor index; SL: seedling length; SDW: seedling dry weight.

Table S2. Analysis of variance for the effects of storage duration, seed moisture content, storage temperature, and plant species, and their interactions on oxidative products and antioxidant and non-antioxidant enzyme activity

Source of variation	DF	Respiration rate	EC	pH	MDA	H_2O_2	$O_2^{\bullet -}$	SOD	CAT	APX	Tocopherol
Storage duration (D)	3	0.024**	124.91**	26.94**	254.26**	195.98**	48.63**	98.97**	34.4**	21.83**	247229.83**
Moisture content (M)	3	0.0269**	78.93**	16.95**	118.2**	144.27**	37.32**	47.18**	23.79**	10.89**	136141.71**
Storage temperature (T)	2	0.0096**	38.92**	7.42**	36.71**	77.66**	17.16**	24.7**	11.74**	5.92**	56796.51**
Plant species (S)	1	0.0195**	134.47**	16.25**	322.33**	315.58**	47.54**	41.5**	24.31**	38.97**	277770.83**
D×M	9	0.000176**	0.82**	0.09**	9.97**	12.91**	0.36**	0.2**	0.4**	0.06**	1596.8**
D×T	6	0.0011**	4.78**	0.86**	7.52**	16.26**	1.97**	2.82**	1.16**	0.44**	7287.11**
D×S	3	0.000031**	4.52**	0.71**	78.99**	13.36**	0.89**	0.9**	0.31**	1.66**	2899.44**
M×T	6	0.00005*	0.3n.s	0.07**	3.62*	7.27*	0.13**	0.09**	0.05n.s	0.11**	1171.57**
M×S	3	0.000221**	1.64**	0.15**	12.9**	12.49*	0.28**	0.14**	0.39**	0.27**	1083.2**
T×S	2	0.000008n.s	0.23n.s	0.02n.s	2.16n.s	5.05n.s	0.03**	0.04*	0.0029n.s	0.05**	1707.69**
D×M×T	18	0.000047**	0.76**	0.08**	4.91**	9.72**	0.1**	0.12**	0.07**	0.04**	245.12**
D×M×S	9	0.000229**	1.46**	0.28**	12.17**	21.09**	0.2**	0.25**	0.55**	0.01**	1486.3**
D×T×S	6	0.000015n.s	0.66*	0.05**	5.03**	14.43**	0.21**	0.04**	0.13**	0.05**	313.43**
M×T×S	6	0.000019n.s	0.72*	0.07**	5.1**	13.16**	0.08**	0.08**	0.07*	0.01**	95.14**
D×M×T×S	18	0.000047**	0.51*	0.05**	3.89**	7.79**	0.13**	0.12**	0.05*	0.08**	180.86**
Error	192	0.00002	0.29	0.02	1.3	3.23	0.005	0.01	0.03	0.001	9.02
CV		3.63	3.19	2.6	1.22	1.54	1.2	1.42	3.69	1.01	1.31

ns, *, and **, not significant and significant at 5 and 1% probability levels, respectively. Each dependent variable was analyzed separately using ANOVA; each column presents the corresponding ANOVA results for the respective trait. Df: degree of freedom; EC: electrical conductivity; MDA: malondialdehyde; H_2O_2: hydrogen peroxidase; $O_2^{\bullet -}$: superoxide; SOD: superoxide dismutase; CAT: catalase; APX: ascorbate peroxidase.

Table S3. Analysis of variance for the effects of storage duration, seed moisture content, storage temperature, and plant species, and their interactions on seed storage compounds

Source of variation	DF	Oil	LNA	LA	OA	PA	SA	TSS	Sucrose	Starch	Glucose	Fructose	Soluble protein	Total free amino acid
Storage duration (D)	3	338**	503.42**	165.27**	196.46**	76.74**	2.85**	263.77**	113.08**	99.76**	67.76**	166.38**	68.06**	426.64**
Moisture content (M)	3	149.48**	248.51**	157.67**	109.04**	50.79**	1.86**	125.09**	69.4**	55.95**	42.35**	101.4**	33.16**	301.93**
Storage temperature (T)	2	90.37**	125.18**	62.04**	47.49**	20.59**	0.88**	56.88**	31.58**	27.76**	21.53**	41.6**	17.41**	126.02**
Plant species (S)	1	226.97**	441.07**	388.25**	236.02**	78.41**	2.52**	122.21**	58.39**	112.34**	34.07**	162.71**	47.03**	617.18**
D×M	9	10.61**	3.07**	1.71**	0.36**	1.21**	0.01**	1.56**	0.12**	0.72**	0.19**	1.95**	0.21**	15.73**
D×T	6	16.99**	16.32**	7.07**	5.4**	2.66**	0.1**	7.51**	3.61**	3.44**	2.4**	5.09**	1.95**	22.04**
D×S	3	10.91**	3.95**	5.38**	0.2**	3.17**	0.06**	4.02**	0.94**	0.41n.s	0.3**	6.87**	0.73**	14.57**
M×T	6	7.12**	2.69**	0.32*	0.56**	0.49**	0**	0.57**	0.1**	0.47n.s	0.05**	1.35**	0.17**	9.14**
M×S	3	2.37n.s	1.63n.s	6.06**	0.2**	0.62**	0**	0.06n.s	0.78**	1.45**	0.03n.s	4.37**	0.21**	20.78**
T×S	2	5.38*	1.8n.s	2.04**	0.06*	0.11n.s	0.01**	0.17n.s	0.05n.s	0.62n.s	0.02n.s	0.44n.s	0.02n.s	4.48n.s
D×M×T	18	5.05**	3.55**	0.3**	0.24**	0.62**	0.01**	0.69**	0.2**	0.74**	0.06**	1.11**	0.09**	11.77**
D×M×S	9	5.55**	3.47**	0.22n.s	0.56**	1.33**	0.01**	0.89**	0.5**	1.41**	0.17**	2.6**	0.09*	22.53**
D×T×S	6	4.18*	3.21**	0.3*	0.27**	0.36**	0**	0.53**	0.14**	0.51n.s	0.01n.s	0.79*	0.04n.s	11**
M×T×S	6	2.25n.s	2.73**	0.12n.s	0.18**	0.46**	0**	0.2n.s	0.13**	0.56n.s	0.03*	1.19**	0.08n.s	9.72**
D×M×T×S	18	3.34**	3.36**	0.24**	0.14**	0.16**	0**	0.29**	0.21**	0.72**	0.07**	0.62*	0.07*	5.76**
Error	192	1.6	0.8	0.11	0.01	0.04	0.0007	0.13	0.02	0.28	0.01	0.35	0.04	2.29
CV		2.49	1.76	3.2	1.01	2.9	2.37	1.3	1.16	3.37	1.7	2.5	3.01	1.65

ns, *, and **, not significant and significant at 5 and 1% probability levels, respectively. Each dependent variable was analyzed separately using ANOVA; each column presents the corresponding ANOVA results for the respective trait. Df: degree of freedom; LNA: linoleic acid; LA: linolenic acid; OA: oleic acid; PA: palmitic acid; SA: stearic acid; TSS: total soluble sugar.

Table S4. LogWorth values indicating the statistical significance of storage factors and their interactions for seed quality of *L. iberica* and *L. royleana*

GR			MGT			SL			SDW			VI		
**Source**	**Logworth**	***P*-value**	**Source**	**Logworth**	***P*-value**	**Source**	**Logworth**	***P*-value**	**Source**	**Logworth**	***P*-value**	**Source**	**Logworth**	***P*-value**
(M)	112.496	0.000001	(T)	62.810	0.000001	(M)	154.343	0.000001	(M)	139.023	0.000001	(M)	180.212	0.000001
(T)	98.118	0.000001	(M)	55.882	0.000001	S(D)	117.954	0.000001	(D)	132.318	0.000001	(D)	171.348	0.000001
(D)	81.781	0.000001	(D)	38.113	0.000001	(T)	117.512	0.000001	(T)	103.027	0.000001	(T)	144.233	0.000001
(S)	60.424	0.000001	(S)	34.232	0.000001	(S)	102.939	0.000001	(S)	80.567	0.000001	(S)	112.800	0.000001
(D)* (M)	5.148	0.00001	(D)*(T)*(S)	13.408	0.000001	(M)*(S)	7.303	0.000001	(M)*(T)	5.891	0.000001	(M)*(S)	4.055	0.00009
(D)*(M)*(T)*(S)	1.640	0.02292	(D)*(T)	13.252	0.000001	(D)*(M)	6.478	0.000001	(M)*(S)	3.708	0.00020	(D)*(M)	3.072	0.00085
(M)*(T)	1.006	0.09871	(T)*(S)	8.937	0.000001	(D)*(M)*(T)*(S)	2.297	0.00505	(M)*(T)*(S)	2.725	0.00188	(D)*(S)	2.302	0.00498
(D)*(M)*(S)	0.559	0.27636	(D)*(S)	8.572	0.000001	(D)*(T)	1.203	0.06272	(D)*(T)*(S)	2.683	0.00207	(D)*(T)	2.032	0.00930
(M)*(T)*(S)	0.516	0.30489	(D)*(M)	1.822	0.01505	(M)*(T)	0.726	0.18786	(D)*(M)*(T)*(S)	2.509	0.00310	(D)*(M)*(T)*(S)	1.387	0.04106
(D)*(T)	0.468	0.34023	(D)*(M)*(T)*(S)	1.490	0.03238	(T)*(S)	0.720	0.19045	(D)*(M)	2.333	0.00465	(T)*(S)	1.259	0.05512
(D)*(M)*(T)	0.395	0.40260	(D)*(M)*(T)	0.883	0.13093	(D)*(M)*(T)	0.612	0.24425	(D)*(M)*(S)	2.131	0.00740	(D)*(T)*(S)	1.000	0.09992
(M)*(S)	0.315	0.48386	(M)*(S)	0.880	0.13194	(M)*(T)*(S)	0.341	0.45627	(D)*(T)	1.258	0.05523	(M)*(T)*(S)	0.679	0.20931
(D)*(T)*(S)	0.282	0.52209	(D)*(M)*(S)	0.727	0.18768	(D)*(M)*(S)	0.300	0.50150	(T)*(S)	0.727	0.18747	(M)*(T)	0.523	0.29993
(T)*(S)	0.164	0.68534	(M)*(T)*(S)	0.239	0.57722	(D)*(S)	0.149	0.70980	(D)*(S)	0.695	0.20164	(D)*(M)*(S)	0.505	0.31253
(D)*(S)	0.062	0.86682	(M)*(T)	0.055	0.88206	(D)*(T)*(S)	0.141	0.72305	(D)*(M)*(T)	0.098	0.79872	(D)*(M)*(T)	0.442	0.36121

LogWorth = −log10(P). Higher LogWorth values indicate stronger statistical evidence against the null hypothesis (i.e., smaller P-values) and should not be interpreted as measures of effect size or the magnitude of biological effects. : moisture content; T: storage temperature; D: storage duration; S: plant species; GR: germination rate; MGT: mean germination time; VI: vigor index; SL: seedling length; SDW: seedling dry weight

Table S5. LogWorth values indicating the statistical significance of storage factors and their interactions for respiration rate and oxidative products of *L. iberica* and *L. royleana*

	RS				EC			pH		MDA			H_2O_2			$O_2{\bullet}^-$	
**Source**	**Logworth**	***P*-value**	**Source**	**Logworth**	***P*-value**	**Source**	**Logworth**	***P*-value**	**Source**	**Logworth**	***P*-value**	**Source**	**Logworth**	***P*-value**	**Source**	**Logworth**	***P*-value**
(M)	108.870	0.000001	(D)	86.675	0.000001	(M)	123.658	0.000001	(D)*(S)	31.706	0.000001	(M)	25.535	0.000001	(M)	140.783	0.000001
(D)	82.876	0.000001	(M)	80.618	0.000001	(D)	122.000	0.000001	(D)	29.867	0.000001	(D)	25.000	0.000001	(D)	119.841	0.000001
(T)	77.598	0.000001	(S)	56.690	0.000001	(T)	94.212	0.000001	(S)	23.534	0.000001	(S)	17.547	0.000001	(T)	103.919	0.000001
(S)	67.479	0.000001	(T)	54.646	0.000001	(S)	64.956	0.000001	(M)	0.330	0.00805	(T)	17.215	0.000001	(S)	95.133	0.000001
(M)*(T)	4.770	0.00002	(D)*(M)	5.946	0.000001	(D)*(M)*(S)	11.957	0.000001	(T)	0.238	0.00469	(M)*(T)*(S)	4.704	0.00002	(D)*(M)*(T)*(S)	6.673	0.000001
(D)*(M)*(T)*(S)	3.043	0.00091	(M)*(T)*(S)	5.825	0.000001	(D)*(M)*(T)*(S)	7.987	0.000001	(D)*(M)*(S)	0.222	0.00289	(D)*(M)*(T)	4.477	0.00003	(D)*(S)	5.875	0.000001
(D)*(M)*(S)	1.921	0.01198	(D)*(T)	4.196	0.00006	(D)*(T)*(S)	5.900	0.000001	(D)*(M)	0.157	0.00198	(D)*(M)*(S)	4.336	0.00005	(M)*(T)	5.234	0.00001
(D)*(S)	1.760	0.01738	(D)*(M)*(S)	4.020	0.00010	(M)*(T)*(S)	5.611	0.000001	(D)*(T)*(S)	0.153	0.00308	(D)*(T)*(S)	3.404	0.00039	(D)*)	4.029	0.00009
(D)*(M)*(T)	1.533	0.02928	(D)*(M)*(T)	3.634	0.00023	(D)*(S)	3.761	0.00017	(D)*(T)	0.103	0.00268	(D)*(S)	0.851	0.01081	(D)*(T)	1.136	0.07304
(D)*(M)	1.225	0.05963	(D)*(T)*(M)*(S)	2.549	0.00282	(M)*(S)	3.330	0.00047	(M)*(T)*(S)	0.092	0.01974	(M)*(S)	0.786	0.03374	(M)*(T)*(S)	0.862	0.13735
(M)*(S)	1.077	0.08373	(D)*(S)	0.697	0.20068	(T)*(S)	1.673	0.02123	(D)*(M)*(T)*(S)	0.088	0.04611	(D)*(M)*(T)*(S)	0.742	0.04094	(M)*(S)	0.743	0.18079
(M)*(T)*(S)	0.889	0.12916	(M)*(S)	0.487	0.32588	(M)*(T)	0.693	0.20266	(M)*(S)	0.078	0.83520	(T)*(S)	0.506	0.31220	(D)*(M)*(T)	0.711	0.19451
(D)*(T)*(S)	0.531	0.29433	(M)*(T)	0.414	0.38561	(D)*(M)*(T)	0.264	0.54394	(M)*(T)	0.068	0.85428	(D)*(M)	0.340	0.45671	(T)*(S)	0.100	0.79353
(D)*(T)	0.149	0.70933	(T)*(S)	0.251	0.56069	(D)*(M)	0.177	0.66496	(T)*(S)	0.054	0.88299	(D)*(T)	0.295	0.50681	(D)*(T)*(S)	0.030	0.93302
(T)*(S)	0.000	0.94000	(D)*(T)*(S)	0.089	0.81403	(D)*(T)	0.086	0.81944	(D)*(M)*(T)	0.001	0.99766	(M)*(T)	0.072	0.84688	(D)*(M)*(S)	0.018	0.95892

LogWorth = −log10(P). Higher LogWorth values indicate stronger statistical evidence against the null hypothesis (i.e., smaller P-values) and should not be interpreted as measures of effect size or the magnitude of biological effects. : moisture content; T: storage temperature; D: storage duration; S: plant species; RS: respiration rate; EC: electrical conductivity; MDA: malondialdehyde; H_2O_2: hydrogen peroxidase; $O_2{\bullet}^-$: superoxide

Table S6. LogWorth values indicating the statistical significance of storage factors and their interactions for antioxidant enzyme activities of *L. iberica* and *L. royleana*

SOD			CAT			APX			Tocopherol		
**Source**	**Logworth**	***P*-value**	**Source**	**Logworth**	***P*-value**	**Source**	**Logworth**	***P*-value**	**Source**	**Logworth**	***P*-value**
(D)	158.667	0.000001	(D)	121.477	0.000001	(D)	163.872	0.000001	(M)	177.612	0.000001
(M)	142.374	0.000001	(M)	116.422	0.000001	S)	153.206	0.000001	(D)	173.157	0.000001
(T)	111.776	0.000001	(T)	85.863	0.000001	(M)	151.166	0.000001	(S)	156.275	0.000001
(S)	85.606	0.000001	(S)	69.539	0.000001	(T)	120.744	0.000001	(T)	143.332	0.000001
(D)*(S)	13.789	0.000001	(D)*(M)	13.480	0.000001	(D)*(S)	44.603	0.000001	(D)*(M)	26.276	0.000001
(D)*(M)*(T)	2.255	0.00555	(D)*(M)*(S)	8.421	0.000001	(M)*(S)	11.821	0.000001	(D)*(S)	21.471	0.000002
(M)*(T)	1.470	0.03388	(D)*(T)	6.018	0.000001	(T)*(S)	7.793	0.000001	(M)*(S)	18.158	0.000001
(M)*(T)*(S)	1.254	0.03773	(M)*(T)*(S)	5.100	0.00001	(D)*(M)*(T)*(S)	6.225	0.000001	(M)*(T)	16.504	0.000003
(D)*(T)	1.188	0.03583	(D)*(T)*(S)	3.767	0.00017	(M)*(T)	4.096	0.00008	(D)*(T)	14.959	0.000001
(D)*(M)*(T)*(S)	1.097	0.04006	(D)*(M)*(T)	3.302	0.00050	(D)*(M)*(T)	3.302	0.00050	(T)*(S)	9.308	0.000001
(D)*(M)	0.987	0.10295	(D)*(M)*(T)*(S)	1.905	0.01245	(M)*(T)*(S)	1.530	0.02950	(D)*(M)*(T)*(S)	6.975	0.000001
(D)*(T)*(S)	0.656	0.22086	(D)*(S)	0.583	0.26100	(D)*(M)*(S)	0.201	0.62940	(M)*(T)*(S)	5.994	0.000001
(D)*(M)*(S)	0.531	0.29414	(M)*(S)	0.285	0.51931	(D)*(M)	0.154	0.70163	(D)*(M)*(S)	4.183	0.000070
(M)*(S)	0.230	0.58848	(M)*(T)	0.178	0.66395	(D)*(T)*(S)	0.126	0.74805	D)*(T)*(S)	3.424	0.000380
(T)*(S)	0.040	0.91249	(T)*(S)	0.027	0.94015	(D)*(T)	0.093	0.80744	(D)*(M)*(T)	2.029	0.009361

LogWorth = −log10(P). Higher LogWorth values indicate stronger statistical evidence against the null hypothesis (i.e., smaller P-values) and should not be interpreted as measures of effect size or the magnitude of biological effects. : moisture content; T: storage temperature; D: storage duration; S: plant species; SOD: superoxide dismutase; CAT: catalase; APX: ascorbate peroxidase.

Table S7. LogWorth values indicating the statistical significance of storage factors and their interactions for oil, fatty acids, and total soluble sugar of *L. iberica* and *L. royleana*

Oil			LNA			LA			OA			PA			SA			TSS		
**Source**	**Logworth**	***P*-value**	**Source**	**Logworth**	***P*-value**	**Source**	**Logworth**	***P*-value**	**Source**	**Logworth**	***P*-value**	**Source**	**Logworth**	***P*-value**	**Source**	**Logworth**	***P*-value**	**Source**	**Logworth**	***P*-value**
(D)	100.764	0.000001	(D)	111.669	0.000001	(D)	111.669	0.000001	(D)	111.669	0.000001	(M)	94.506	0.000001	(D)	111.669	0.000001	(D)	121.532	0.000001
(M)	82.052	0.000001	(M)	99.953	0.000001	(M)	99.953	0.000001	(M)	99.953	0.000001	(D)	90.634	0.000001	(M)	99.953	0.000001	(M)	103.793	0.000001
(T)	57.309	0.000001	(S)	76.019	0.000001	(S)	76.019	0.000001	(S)	76.019	0.000001	(T)	69.880	0.000001	(S)	76.019	0.000001	(T)	76.572	0.000001
(S)	46.292	0.000001	(T)	73.255	0.000001	(T)	73.255	0.000001	(T)	73.255	0.000001	(S)	65.187	0.000001	(T)	73.255	0.000001	(S)	54.796	0.000001
(D)*(M)	9.270	0.000001	(D)*(S)	8.204	0.000001	(D)*(S)	8.204	0.000001	(D)*(S)	8.204	0.000001	(D)*(M)*(T)	14.889	0.000001	(D)*(S)	8.204	0.000001	(D)*(M)*(S)	6.414	0.000001
(D)*(M)*(T)	7.276	0.000001	(M)*(T)	1.847	0.01423	(M)*(T)	1.847	0.01423	(M)*(T)	1.847	0.01423	(D)*(M)*(S)	11.147	0.000001	(M)*(T)	1.847	0.01423	(D)*(T(S)	3.993	0.00010
(D)*(T)	5.182	0.00001	(D)*(M)*(T)*(s)	1.554	0.02790	(D)*(M)*(T)*(s)	1.554	0.02790	(D)*(M)*(T)*(s)	1.554	0.02790	(M)*(T)*(S)	5.866	0.000001	(D)*(M)*(T)*(s)	1.554	0.02790	(D)*(M)*(T)	3.102	0.00079
(D)*(T)*(S)	3.056	0.00088	(M)*(T)*(S)	1.092	0.08100	(M)*(T)*(S)	1.092	0.08100	(M)*(T)*(S)	1.092	0.08100	(D)*(T)*(S)	5.462	0.000001	(M)*(T)*(S)	1.092	0.08100	(M)*(T)	2.117	0.00764
(D)*(M)*(S)	2.723	0.00189	(D)*(T)*(S)	0.807	0.15604	(D)*(T)*(S)	0.807	0.15604	(D)*(T)*(S)	0.807	0.15604	(D)*(S)	4.633	0.00002	(D)*(T)*(S)	0.807	0.15604	(D)*(S)	1.176	0.00670
(M)*(T)*S)	2.588	0.00258	(D)*(M)*(S)	0.769	0.17016	(D)*(M)*(S)	0.769	0.17016	(D)*(M)*(S)	0.769	0.17016	(M)*(S)	3.423	0.00038	(D)*(M)*(S)	0.769	0.17016	(M)*(T)*(S)	0.664	0.01681
(M)*(T)	1.125	0.03497	(M)*(S)	0.365	0.43123	(M)*(S)	0.365	0.43123	(M)*(S)	0.365	0.43123	(T)*(S)	1.510	0.03090	(M)*(S)	0.365	0.43123	(D)*(M)*(T)*(S)	0.433	0.02882
(D)*(M)*(T)*(S)	1.036	0.04194	(T)*(S)	0.277	0.52880	(T)*(S)	0.277	0.52880	(T)*(S)	0.277	0.52880	(M)*(T)	1.052	0.03881	(T)*(S)	0.277	0.52880	(M)*(S)	0.083	0.02616
(D)*(S)	0.510	0.30921	(D)*(M)*(T)	0.194	0.64027	(D)*(M)*(T)	0.194	0.64027	(D)*(M)*(T)	0.194	0.64027	(D)*(M)	0.803	0.03647	(D)*(M)*(T)	0.194	0.64027	(D)*(T)	0.080	0.83155
(T)*(S)	0.276	0.52931	(D)*(T)	0.172	0.67307	(D)*(T)	0.172	0.67307	(D)*(T)	0.172	0.67307	(D)*(M)*(T)*(S)	0.682	0.04082	(D)*(T)	0.172	0.67307	(T)*(S)	0.015	0.96691
(M)*(S)	0.096	0.80249	(D)*(M)	0.127	0.74618	(D)*(M)	0.127	0.74618	(D)*(M)	0.127	0.74618	(D)*(T)	0.255	0.55530	(D)*(M)	0.127	0.74618	(D)*(M)	0.011	0.97604

LogWorth = −log10(P). Higher LogWorth values indicate stronger statistical evidence against the null hypothesis (i.e., smaller P-values) and should not be interpreted as measures of effect size or the magnitude of biological effects. M: moisture content; T: storage temperature; D: storage duration; S: plant species; LNA: linoleic acid; LA: linolenic acid; OA: oleic acid; PA: palmitic acid; SA: stearic acid; TSS: total soluble sugar.

Table S8. LogWorth values indicating the statistical significance of storage factors and their interactions for storage compounds of *L. iberica* and *L. royleana*

	Sucrose			Starch			Glucose			Fructose			Soluble protein			Total free amino acid	
**Source**	**Logworth**	***P*-value**	**Source**	**Logworth**	***P*-value**	**Source**	**Logworth**	***P*-value**	**Source**	**Logworth**	***P*-value**	**Source**	**Logworth**	***P*-value**	**Source**	**Logworth**	***P*-value**
(D)	157.389	0.000001	(D)	92.890	0.000001	(M)	185.922	0.000001	(D)	90.818	0.000001	(D)	146.607	0.000001	(D)	51.137	0.000001
(M)	156.206	0.000001	(M)	84.383	0.000001	(D)	185.097	0.000001	(M)	86.004	0.000001	(M)	121.946	0.000001	(M)	48.822	0.000001
(T)	123.775	0.000001	(S)	65.106	0.000001	(T)	151.080	0.000001	(T)	62.939	0.000001	(T)	94.481	0.000001	(S)	37.916	0.000001
(S)	86.320	0.000001	(T)	60.925	0.000001	(S)	108.135	0.000001	(S)	59.536	0.000001	(S)	94.122	0.000001	(T)	33.188	0.000001
(D)*(M)*(S)	6.472	0.000001	(D)*(T)*(S)	2.194	0.00639	(D)*(M)	9.696	0.000001	(D)*(S)	6.769	0.000001	(D)*(S)	12.691	0.000001	(D)*(M)*(T)	8.015	0.000001
(M)*(T)*(S)	6.016	0.000001	(M)*(T)*(S)	2.192	0.00642	(D)*(M)*(S)	6.250	0.000001	(M)*(T(S)	5.853	0.000001	(D)*(M)	9.640	0.000001	(D)*(M)*(S)	6.620	0.000001
(D)*(M)*(T)	4.335	0.00005	(D)*(M)*(S)	2.089	0.00815	(D)*(T)	5.174	0.00001	(M)*(T)	5.279	0.00001	(D)*(T)	5.350	0.000001	(D)*(T)*(S)	4.515	0.00003
(D)*(T)*(S)	4.160	0.00007	(M)*(T)	1.245	0.00683	(D)*(M)*(T)*(S)	3.810	0.00016	(D)*(M)*(T)	4.748	0.00002	(M)*(T)*(S)	5.085	0.00001	(M)*(T)*(S)	4.145	0.00007
(M)*(S)	2.819	0.00152	(D)*(M)*(T)	0.869	0.00518	(M)*(T)	3.404	0.00039	M)*(S)	4.059	0.00009	(D)*(M)*(T)	3.408	0.00039	(M)*(T)	1.592	0.02556
(D)*(M)*(T)*(S)	2.096	0.00802	(D)*(M)	0.712	0.00391	(D)*(T)*(S)	3.249	0.00056	(T)*(S)	3.350	0.00045	(D)*(M)*(S)	1.667	0.02150	(T)*(S)	1.319	0.03799
(D)*(S)	2.018	0.00959	(D)*(S)	0.605	0.02838	(M)*(T)*(S)	2.726	0.00188	(D)*(M(S)	3.130	0.00074	(M)*(T)	1.289	0.03141	(M)*S)	1.250	0.03819
(T)*(S)	1.615	0.02428	(D)*(T)	0.480	0.03093	(D)*(M)*(T)	2.039	0.00913	(D)*(T)*(S)	2.099	0.00796	M)*(S)	1.064	0.03636	(D)*(M)	0.196	0.04124
(M)*(T)	0.682	0.20793	(M)*(S)	0.136	0.03186	(D)*(S)	0.403	0.39536	(D)*(M)	1.570	0.02692	(D)*(T)*(S)	0.588	0.38809	(D)*(S)	0.123	0.04302
(D)*(M)	0.520	0.30201	(D)* (M)*(T)*(S)	0.065	0.04099	(T)*(S)	0.201	0.62988	(D)*(T)	1.083	0.03252	(D)*(M)*(T)*(S)	0.548	0.04313	(D)*(M)*(T)*(S)	0.113	0.04673
(D)* (T)	0.229	0.59030	(T)*(S)	0.035	0.06342	(M)*(S)	0.161	0.69103	(D)*(M)*(T)*(S)	1.056	0.04787	(T)*(S)	0.304	0.49716	(D)*(T)	0.069	0.05292

LogWorth = −log10(P). Higher LogWorth values indicate stronger statistical evidence against the null hypothesis (i.e., smaller P-values) and should not be interpreted as measures of effect size or the magnitude of biological effects. M: moisture content; T: storage temperature; D: storage duration; S: plant species.
